# Comparative efficacy and safety of glucose-lowering drugs in children and adolescents with type 2 diabetes: A systematic review and network meta-analysis

**DOI:** 10.3389/fendo.2022.897776

**Published:** 2022-08-11

**Authors:** Sijia Wu, Yina He, Yutong Wu, Yiman Ji, Lei Hou, Xinhui Liu, Yilei Ge, Yuanyuan Yu, Yifan Yu, Yun Wei, Fengtong Qian, Qingxin Luo, Yue Feng, Yiping Feng, Jiongjiong Wang, Meiling Huo, Hongkai Li, Fuzhong Xue, Yunxia Liu

**Affiliations:** ^1^ Department of Biostatistics, School of Public Health, Cheeloo College of Medicine, Shandong University, Jinan, China; ^2^ Institute for Medical Dataology, Cheeloo College of Medicine, Shandong University, Jinan, China; ^3^ Qilu Children’s Hospital of Shandong University, Jinan, China

**Keywords:** systematic review & meta-analysis, glucose-lowering drugs, frequentist network meta- analysis, adolescents, glycosylated hemoglobin A1c, type 2 diabetes

## Abstract

**Objective:**

Type 2 diabetes is more common in adults, but is becoming the major concern in children and adolescent recently. This study aimed to provide additional pharmaceutical management for children and adolescents with type 2 diabetes by assessing the efficacy and safety of several glucose-lowering drugs.

**Methods:**

Searches were performed in PubMed, Medline, Ovid, Cochrane Controlled Register of Trials (CENTRAL), and ClinicalTrials.gov that reported the efficacy and safety of drugs for children and adolescents with type 2 diabetes. Pooled effects were calculated by frequentist fixed effects network meta-analyses and additive network meta-analyses.

**Results:**

A total of 12 trials assessing eight glucose-lowering drugs were included, which compose of seven trials with monotherapy and five trials with combination therapies. Network meta-analysis results showed compared to placebo, saxagliptin+metformin (mean difference (MD) -1.91% [-2.85%, -0.97%]), liraglutide+metformin (MD -1.45% [-1.65%, -1.26%]), and liraglutide (MD -0.90% [-1.35%, -0.45%]) were the top 3 drugs that significantly reduced hemoglobin A1c (HbA1c). Sitagliptin+metformin, dapagliflozin, exenatide-2mcg, linagliptin-5mg, metformin, exenatide-5/10mcg, glimepiride, and sitagliptin also showed significant reduction in HbA1c. There were no significant differences between treatments in the incidence of adverse events, except that liraglutide+metformin had significant adverse effect such as abdominal pain. In addition, dapagliflozin, sitagliptin+metformin, and saxagliptin+metformin showed better efficacy compared with FDA-approved drugs.

**Conclusions:**

The top 10 treatments of type 2 diabetes in children and adolescents aged 10–17 years were saxagliptin+metformin, liraglutide+metformin, liraglutide, dapagliflozin, exenatide–2 mcg, sitagliptin+metformin, linagliptin–5 mg, linagliptin–1 mg, metformin, and exenatide–5/10 mcg.

**Systematic Review Registration:**

https://www.crd.york.ac.uk/prospero/display_record.php?RecordID=284897, identifier CRD42021284897.

## 1 Introduction

Type 2 diabetes has become a major concern in children and adolescents recently (1). Family history and genetics, lifestyle including diet, exercise, and weight management play important roles in type 2 diabetes development. From 2007 to 2017, the incidence of type 2 diabetes among children and adolescents increased significantly, with an overall annual increase of 4.8% (2, 3). Epidemiological studies have showed that in the US, the estimated prevalence of diabetes among children and adolescents increased significantly for type 2 diabetes (4). In Asian, countries, the incidence of type 2 diabetes in youth is even higher than that of type 1 diabetes (5). studies indicated that over 85% of children with type 2 diabetes are overweight or obese at diagnosis (6, 7). More importantly, among people diagnosed with type 2 diabetes in childhood, the risk of complications including hyperglycemia, hypertension, and dyslipidemia increased steadily over time (8, 9).

Management of children and adolescents with type 2 diabetes typically includes healthy lifestyle modification and pharmaceutical intervention. So far, the US Food and Drug Administration (FDA) has approved four medications for the treatment of children and adolescents with type 2 diabetes: metformin, liraglutide, exenatide, and insulin; however, all of them have some adverse reactions. Metformin is a first-line pharmaceutical agent to lower high blood sugar levels for children and adolescents with type 2 diabetes, especially for overweight patients (10). Metformin works by lowering the amount of glucose absorbed from intestines, decreasing how much glucose is made in the liver and improving insulin sensitivity. Gastrointestinal intolerance, including abdominal discomfort, nausea, and diarrhea, is the major concern of its safety (11). Both liraglutide and exenatide are glucagon-like peptide (GLP-1) analogue agents. Liraglutide, which was approved by the FDA in 2019, is effective in reducing blood glucose, while avoiding hypoglycemia and weight gain (12). It has been demonstrated that the addition of liraglutide to metformin in adolescents can effectively improve glucose control (13). Exenatide was approved by the FDA in July 2021 for the treatment alone or in combination with metformin. It is a once-weekly injection, which has positive effects on glucose control, weight management, and heart health (14). Similarly, the most commonly reported adverse events of GLP-1 analogue agents were notably nausea, vomiting, and diarrhea. Glimepiride is a potent sulfonylurea and can be used alone, in combination with other glucose-lowering agents, or with diet and exercise to improve blood sugar control in patients with type 2 diabetes (15). It has been demonstrated that glimepiride can reduce HbA1c similarly to metformin with greater weight gain and comparable safety (16). It’s worth noting that the last three drugs, all can be used in combination with metformin and that the primary purpose of all medications is to lower blood sugar levels. Therefore, it is urgent to develop pharmaceutical management options for children and adolescents with type 2 diabetes to better control glucose level, reduce the occurrence of adverse reactions and prevent long-term and even life-threatening complications.

Compared to wide optional agents for adults, children and adolescents have limited options, as previously mentioned. Given the increasing numbers of children and adolescents with type 2 diabetes, more optional pharmaceutical agents for them will afford more beneficial effects on glucose control, reduce the occurrence of adverse reactions and prevent the complications associated with type 2 diabetes. In addition, there are few head-to-head drug comparation for children and adolescents with type 2 diabetes. Therefore, this systematic review and network meta-analysis aimed to show the current ranking of medications used for the treatment of children and adolescents with type 2 diabetes and provide additional pharmaceutical management by assessing the efficacy and safety of several glucose-lowering drugs.

## 2 Material and methods

### 2.1 Search strategy

We registered the protocol in PROSPERO (CRD42021284897), and report our methods and results according to the Preferred Reporting Items for Systematic Reviews and Meta-analyses extension statement for network meta-analyses. We searched PubMed, Medline, Ovid, CENTRAL, and ClinicalTrials.gov from inception through 15 September 2021 without language restrictions with CADTH filters. The detailed search strategies are given in the **Supplementary Material S1**. Moreover, the reference lists from the retrieved articles, systematic reviews, and meta-analyses were checked to search for further releva0nt studies.

### 2.2 Selection criteria and eligibility criteria

Inclusion criteria were shown as follows: 1) Study design: randomized clinical trials (RCTs) were included; 2) Population: studies were conducted among populations aged less than 17 years with type 2 diabetes, studies including patients with other chronic diseases were excluded; 3) Intervention: studies containing two or more different drugs (including placebo) were eligible for inclusion; 4) Outcomes: studies that reported both the change of HbA1c and the percentage of patients with adverse events after intervention compared with the control group were included. In order to further evaluate the efficacy and safety of drugs, we also considered the change in fasting plasma glucose (FPG) from baseline, the percentage of patients achieving HbA1c goals of ≤ 6.5% and < 7% as secondary efficacy outcomes, as well as gastrointestinal disorders, hyperglycemia, and hypoglycemia as secondary safety outcomes.

Records identified in the updated search were screened for eligibility by two independent reviewers (XL and YG) and any disagreements were resolved through discussion with another reviewer (FQ).

### 2.3 Data extraction

For each eligible study, two reviewers (Sijia Wu and Yina He) independently extracted the following variables: study characteristics (year of publication, country, intervention duration), study design (RCTs), population characteristics (setting, sample size, demographic characteristics, diagnostic criteria for type 2 diabetes), interventions (drug name, dose), and outcomes (primary outcomes and secondary outcomes). The reviewers resolved disagreements through discussion or consultation with a third reviewer (Yutong Wu). Finally, all extracted information was stored in an excel spreadsheet.

### 2.4 Risk of bias assessment

Two reviewers (Sijia Wu and Yina He) independently assessed the risk of bias of included studies using the risk of bias assessment tool from the Cochrane Collaboration, including random sequence generation, allocation concealment, blinding, missing outcome data, and selective reporting of outcomes (17). Each domain was judged as low, unclear, or high risk of bias. Any disagreements were adjudicated by a third reviewer (Yutong Wu).

### 2.5 Data synthesis

Initially, we explored the transitivity assumption of the network meta-analysis by comparing the distributions of potential effect modifiers across treatment comparisons (age, baseline HbA1c level, baseline weight, and baseline BMI). We performed frequentist network meta-analysis and calculated the MD and its 95% confidence interval (CIs) of changes in HbA1c/FPG levels, as well as the odds ratio (OR) and its 95% CIs of the dichotomous outcomes. For quantification of heterogeneity, Cochran’s Q statistic was considered. We decided whether to use a fixed effects model or a random effects model according to the Cochran’s Q statistic. We assessed consistency in networks both locally by comparing the direct with indirect evidence and, globally, with the design-by-treatment interaction model (18, 19). Treatment ranking was calculated according to P-scores, which were based solely on the point estimates and standard errors of the network meta-analysis estimates. These scores measure the extent of certainty that one treatment is better than another and are the average of all competitive treatments (20). The presence of publication bias was assessed by funnel plots. Additionally, Begg’s and Egger’s regression tests were performed to detect small study effects (21).

Since most of the interventions or control groups included in the study were combinations of other treatments or had common components, we also used an additive network meta-analysis model in addition to the fixed effect model commonly used in the past. The additive model assumes that the effect of a treatment combined of two components A and B is the sum of the effects of A and B, which implies that in comparisons equal components cancel out, then the influence of individual components can be evaluated (22).

We also planned to perform sensitivity analysis and subgroup analysis. The first sensitivity analysis used only studies with a low risk of bias. Meanwhile, we also used Bayesian fixed effects network meta-analysis as another sensitivity analysis to verify our results. If new problems were found in subsequent analyses, more sensitivity analyses would be performed. We found that all of the drugs compared in the study were monotherapy or monotherapy plus metformin, so in the subgroup analysis, all treatments were divided into monotherapy and combination groups and compared the results with previous results to determine whether they were consistent.

## 3 Results

### 3.1 Baseline characteristics

**Figure 1:**
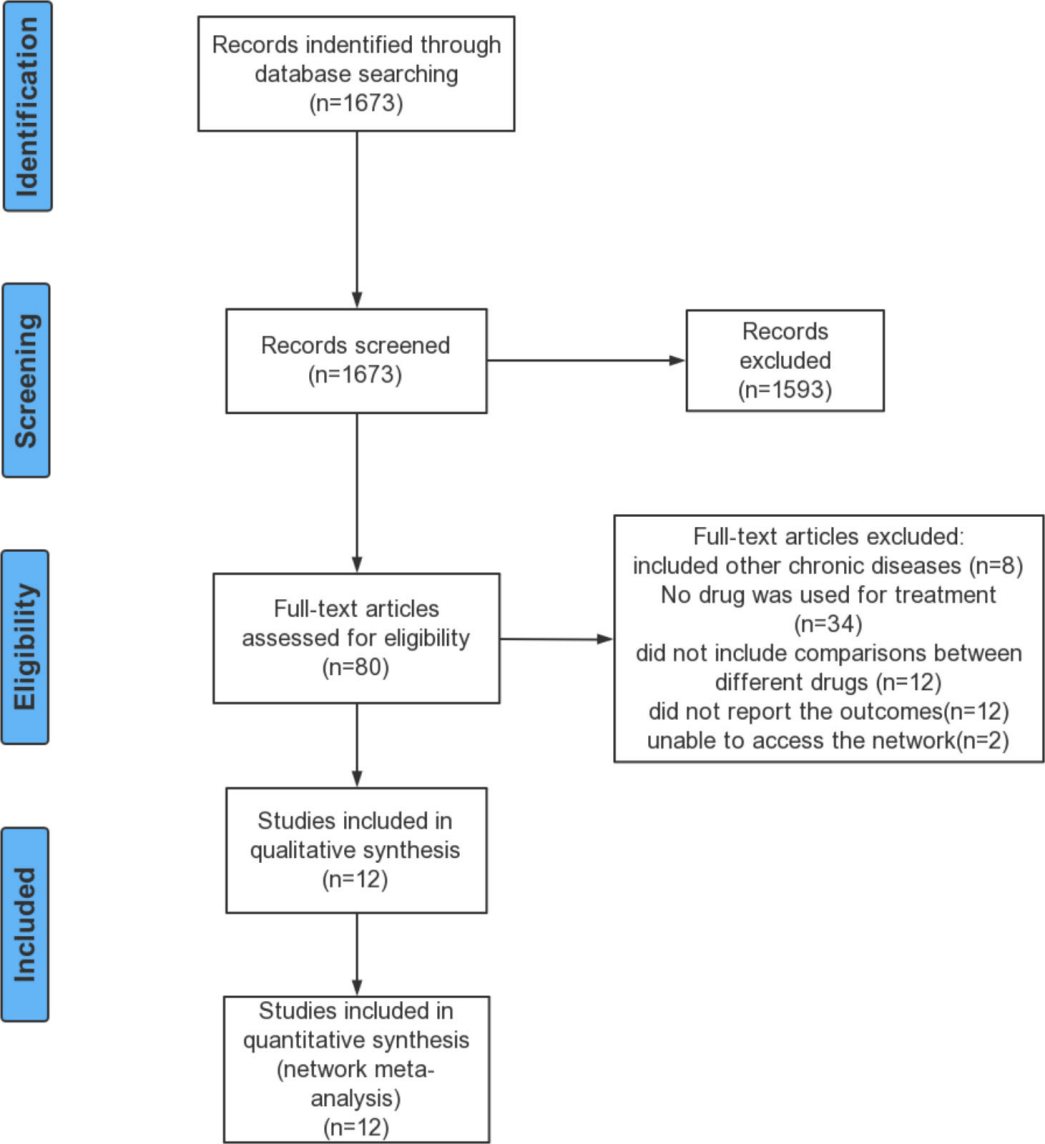
Flow Diagram.

A total of 12 studies (1,237 patients) were included in the systematic review and network meta-analysis, including eight published articles (16, 23–29) and four clinical trials. The detailed selection process was shown in **Figure 1**. The characteristics of the included studies are presented in **Table 1**. The study period of all studies was less than 6 months. Ten studies were double-blind, one was single-blind and one was quadruple. Ten studies provided the mean age of patients, ranging from 13.6 to 16 years. Seven studies provided HbA1c levels at baseline of the patients, ranging from 7.5% to 9%. Eight studies provided the mean weight at baseline of patients ranging from 79.8 kg to 114.2 kg and the mean BMI at baseline ranging from 30 kg/m^2^ to 40 kg/m^2^. Based on the distribution of potential effect modifiers (age, baseline HbA1c level, baseline weight and baseline BMI) in all treatment comparisons, the levels of potential effect modifiers were consistent in all studies except for Klein DJ study’s (24) weight and BMI (Figures a1–a4). Further research would be carried out in the sensitivity analysis.

**Table 1:** Characteristic of included studies.

Author/NCT number	Blinding setting	Study period(weeks)	Interventions	Sample N/F/M	Mean age(years)	BaselineHbA1c(%)	BaselineWeight(kg)	BaselineBMI(kg/m^2^)
NCT00658021	double blind	28	Exenatide –5mcg	42/31/11	14 ± 1.91	6.5-10.5	–	–
			Exenatide–10 mcg	38/22/16			–	–
			Placebo	42/29/13			–	–
NCT01204775	double blind	16	Saxagliptin +Metformin	4/1/3	10-17	7-10.5	–	–
			Placebo	4/3/1			–	–
NCT01434186	double blind	16	Saxagliptin+Metformin	4/4/0	10-17	7-10.5	–	–
			Metformin	2/2/0			–	–
NCT01554618	double blind	24	Exenatide–2 mcg	58/31/27	15.1 ± 1.84	6.5-12	–	–
			Placebo	24/17/7			–	–
Jones KL, 2002 (25)	double blind	8	Metformin	42/30/12	13.9 ± 1.8	8.3 ± 1.3	92.8 ± 31.8	34.2 ± 10.6
			Placebo	40/27/13	13.6 ± 1.8	9.0 ± 1.4	90.3 ± 38.1	33.9 ± 12.7
Gottschalk M, 2007 (16)	single blind	24	Glimepiride	132/88/44	13.8 ± 2.3	8.53 ± 1.58	82.60 ± 25.60	31.57 ± 8.48
			Metformin	131/87/44			83.83 ± 27.47	31.60 ± 8.17
David J Klein, 2014 (24)	double blind	5	Liraglutide	14/9/5	14.4	8.3	112.7	40.0
			Placebo	7/5/2	15.6	7.8	114.2	39.9
William V Tamborlane, 2018 (23)	double blind	12	Linagliptin–1 mg	10/4/6	14.0 ± 1.9	7.86 ± 0.95	79.8 ± 22.2	30.3 ± 6.8
			Linagliptin–5 mg	14/9/5				
			Placebo	15/8/7				
Tamborlane WV, 2019 (26)	double blind	26	Liraglutide+Metformin	66/41/25	14.57 ± 1.72	7.78 ± 1.34	91.5 ± 26.8	33.9 ± 9.25
			Metformin	68/42/26				
Tamborlane WV, 2022 (29)	double blind	24	Dapagliflozin	39/24/15	16.0 ± 3.3	6.5-11	90.7 ± 28.5	32.4 ± 8.1
			Placebo	31/19/12				
Jalaludin MY, 2022 (27)	quadruple	20	Sitagliptin+Metformin	62/41/21	14.4 ± 1.9	8.0 ± 1.1	81.9 ± 25.4	31.2 ± 8.1
			Metformin	62/40/22			79.8 ± 24.8	30.6 ± 8.5
			Sitagliptin+MetforminXR	45/32/13			81.9 ± 25.4	31.2 ± 8.1
			MetforminXR	51/32/19			79.8 ± 24.8	30.6 ± 8.5
Shankar RR, 2022 (28)	double blind	20	Sitagliptin	95/54/41	14.0 ± 2.0	7.5 ± 1.04	89.1 ± 25.3	33.3 ± 7.7
			Placebo	95/61/34			81.9 ± 24.8	31.2 ± 7.7

Except where indicated, data are presented as mean ± SD or minimum–maximum.

N/F/M, Number Analyzed/Female/Male; BMI, body mass index.

### 3.2 Risk of bias assessment

Risk of bias assessment of the included studies was shown in **Supplemental Material S2**. One study (26) was judged to have a high risk of bias in measurement of the outcome. Two studies (16, 29) demonstrated an unclear risk of bias and nine (23–25, 27, 28, NCT01204775, NCT01554618, NCT01434186, and NCT00658021) showed a low risk of bias.

### 3.3 Network meta-analysis

**Figure 2:**
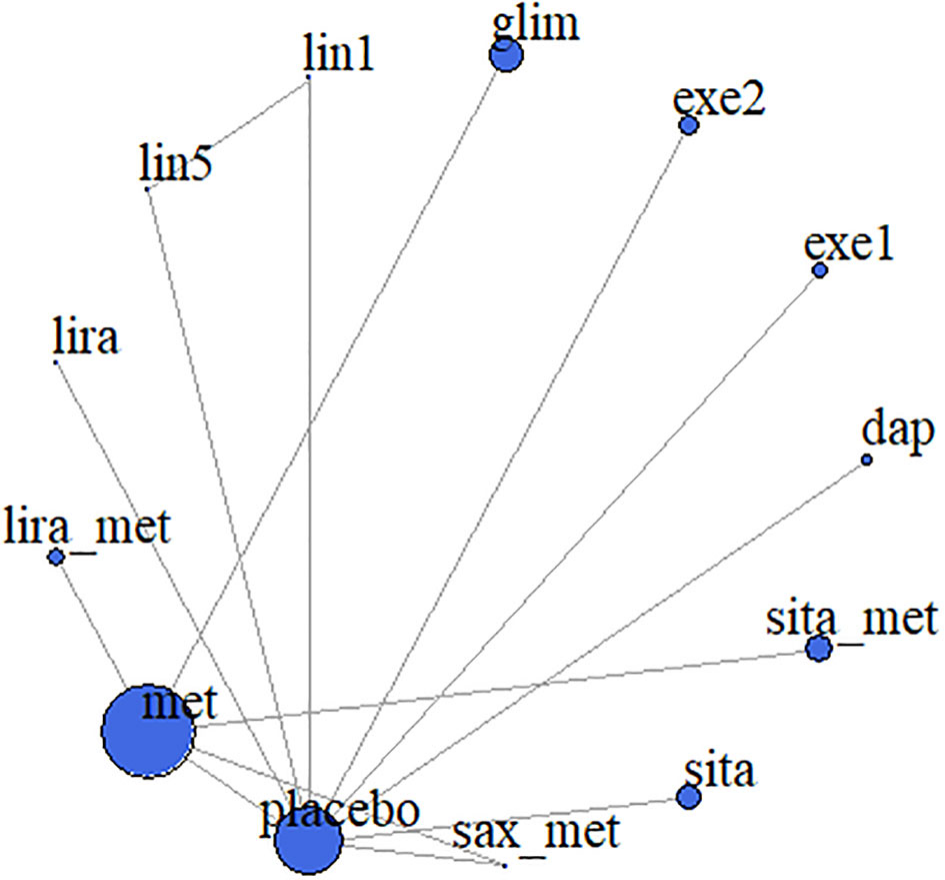
Meta-analysis networks for change in HbA1c. Each circle indicates a treatment node. Lines connecting 2 nodes represent direct comparisons between 2 treatments. The size of the nodes is proportional to the number of trials evaluating each treatment. The thickness of the lines is proportional to the number of trials directly comparing the 2 connected treatments. met, metformin; lira_met, liraglutide+metformin; lira, liraglutide; lin5, linagliptin-5 mg; lin1, linagliptin-1 mg; glim, glimepiride; exe1, exenatide-2 mcg; exe2, exenatide-5/10 mcg; dap, dapagliflozin; sita_placebo, sitagliptin+placebo; sita_met, sitagliptin_metformin; sax_met, saxagliptin+metformin.

We included eight drugs (dapagliflozin, exenatide, sitagliptin, metformin, saxagliptin, linagliptin, glimepiride, and liraglutide) and their combinations in our network meta-analysis (**Figure 2**). In the study, different doses of the same drug were separately analyzed (23), however, in NCT00658021, exenatide (5 mcg) and exenatide (10 mcg) were grouped together when the results were published. Especially, both NCT01760447 metformin and metforminXR are classified as metformin (30). Because Cochran’s Q statistic was too small, there was no evidence of heterogeneity for any outcome (**Table S1**), so that fixed effects model was used in all analysis and the random effects model showed the consistent results with fixed effects model.

#### 3.3.1 Primary outcomes

**Figure 3:**
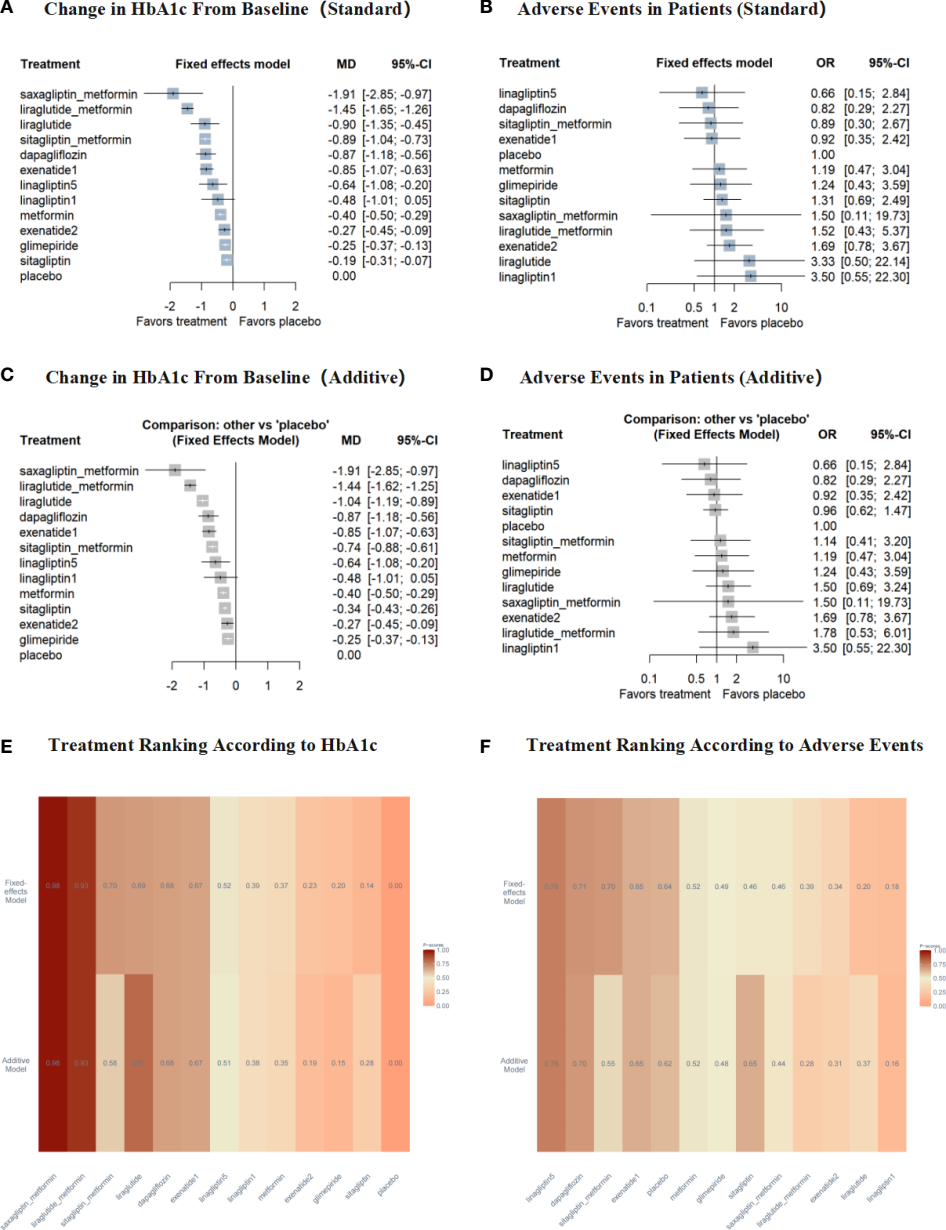
Results for the primary outcomes compared with placebo. Treatments are presented according to their effect estimate compared with placebo. Effect sizes are presented as MD or OR with 95% CI's. MD, mean difference; OR, odds ratio. exenatide1, exenatide-2mcg; exenatide2, exenatide-5/10mcg; linagliptin1, linagliptin-1mg; linagliptin5, linagliptin-5mg. **(A)** the change in HbA1c from baseline of fixed effects model; **(B)** adverse events of patients of fixed effects model; **(C)** the change in HbA1c from baseline of additive model; **(D)** adverse events of patients of additive model; **(E)** treatment ranking according to HbA1c of both models; **(F)** treatment ranking according to adverse events of both models.

The change of HbA1c from baseline was analyzed in 1,273 patients of 12 studies. In fixed effects model, compared to placebo, the reduction of HbA1c was significantly larger in saxagliptin+metformin (MD -1.91% [-2.85%, -0.97%]), liraglutide+metformin (MD -1.45% [-1.65%, -1.26%]), liraglutide (MD -0.90% [-1.35%, -0.45%]), sitagliptin+metformin (MD -0.89% [-1.04%, -0.73%]), dapagliflozin (MD -0.87% [-1.18%, -0.56%]), exenatide–2 mcg (MD -0.85% [-1.07%, -0.63%]), linagliptin–5 mg (MD -0.64% [-1.08%, -0.20%]), metformin (MD -0.40% [-0.50%, -0.29%]), exenatide–5/10 mcg (MD -0.27% [-0.45%, -0.09%]), glimepiride (MD -0.25% [-0.37%, -0.13%]), and sitagliptin (MD -0.19% [-0.31%, -0.07%]), respectively (**Figure 3A**). In additive network meta-analysis, all results were consistent with the fixed effects model (**Figure 3C**).

Furthermore, saxagliptin+metformin showed the greatest potential as the best intervention to improve HbA1c (P-score = 0.98 in both models), liraglutide+metformin was the second best (P-score = 0.93 in both models) and liraglutide (P-score = 0.69 in fixed effects model; 0.81 in additive model; **Figure 3E**, **Table S2**).

There was no significant difference versus placebo in the incidence of adverse events for all treatments in both models (**Figures 3B, D**). Furthermore, linagliptin–5 mg (P-score = 0.76 in both model), dapagliflozin (P-score = 0.71 in fixed effects model; 0.70 in additive model), and exenatide–2 mcg (P-score = 0.65 in both models) showed better effects than placebo (**Figure 3F**, **Table S3**).

#### 3.3.2 Secondary outcomes

##### 3.3.2.1 Efficacy outcomes

Nine studies involving 960 patients reported change in FPG from baseline. In both models, compared to placebo, liraglutide, dapagliflozin, exenatide–2 mcg, linagliptin–5 mg, metformin, exenatide–5/10 mcg, and sitagliptin+metformin all showed significant reduction in FPG. However, sitagliptin showed different results from the fixed effects model (MD -4.57 [-5.06, -4.08] in fixed effects model; MD 1.50 [0.81, 2.19] in additive model). This might be because the additive model assumed that the effect of the treatment combination is the sum of the effects of its components (22). Furthermore, exenatide–2 mcg showed the greatest potential as the best intervention to improve FPG and sitagliptin+metformin was the second best (Figure a6A, **Table S4**).

Eight studies involving 1,161 patients reported the percentage of patients achieving HbA1c goals of less than 7%. In both models, compared to placebo, liraglutide+metformin, sitagliptin+metformin, metformin, glimepiride, dapagliflozin, and exenatide–2 mcg showed significant improvement in the percentage of HbA1c < 7%. Furthermore, according to P-score, all treatments in this analysis were better than placebo (Figure a6B, **Table S5**).

Three studies involving 392 patients reported the percentage of patients achieving HbA1c goals of less than 6.5%. In both models, no treatments showed significant improvement in the percentage of HbA1 ≤ 6.5%. Furthermore, according to P-score, all treatments in this analysis were better than placebo (Figure a6C, **Table S6**).

##### 3.3.2.2 Safety outcomes

Seven studies involving 998 patients reported the number of patients with hyperglycemia, five studies involving 794 patients reported the number of patients with hypoglycemia, eight studies involving 1,085 patients reported the number of patients with upper abdominal pain, 10 studies involving 1,147 patients reported the number of patients with diarrhea, nine studies involving 907 patients reported the number of patients with vomiting, and seven studies involving 679 patients reported the number of patients with nausea. For all treatments in both models, there was no difference versus placebo in the incidence of these outcomes (Figure a7A–F).

Seven studies involving 979 patients reported the number of patients with abdominal pain. In additive network meta-analysis, liraglutide+metformin (OR 7.84 [1.59, 38.67]) showed significant difference from placebo (Figure a7G). The treatments ranking according to P-score for every safety outcome was shown in **Tables S7–S13**.

### 3.4 Sensitivity analyses and subgroup analyses

Results of two sensitivity analyses were both almost similar to primary outcomes. According to the distribution of potential effect modifiers, we found significant differences in baseline weight and BMI between Klein DJ’s study (24) and the others. Therefore, we performed a third sensitivity analyses of the remaining seven studies. The treatment ranking included in the seven studies was consistent with primary outcomes (**Supplemental Material S4**). In the subgroup analyses of studies, results of monotherapy group were similar to those of the primary outcomes. However, no difference was shown between treatments and placebo in the combination group, but there was a significant difference between treatments and placebo in the primary outcomes (**Supplementary Material S4**).

### 3.5 Publication bias and small study effect

Since the included drugs could not be compared directly and indirectly at the same time, the inconsistency analysis could not be carried out. Through funnel plots, there was no publication bias in this study (Figure a14). Through Egger’s linear regression test and Begg’s rank correlation test, there was no small study bias in this study (**Table S14**).

## 4 Discussion

Metformin combined with diet and exercise has been the first-line clinical treatment for children and adolescents with type 2 diabetes, and liraglutide and exenatide have been approved by the FDA in recent years. There are few published studies on the comparative efficacy and safety of different drugs therapy in children and adolescents with type 2 diabetes.

This study used both fixed effects and additive network meta-analysis model to evaluate the relative effects of eight different drugs on HbA1c level, FPG, patients achieving HbA1c goals of < 7%, patients achieving HbA1c goals of ≤ 6.5% and adverse events in children and adolescents with type 2 diabetes. In particular, we considered common adverse reactions to glucose-lowering drugs, such as hyperglycemia, hypoglycemia, and gastrointestinal disorders (31). From the 12 studies, our network meta-analysis indicated that saxagliptin+metformin, liraglutide+metformin, liraglutide, sitagliptin+metformin, dapagliflozin, exenatide–2 mcg, linagliptin–5 mg, metformin, exenatide–5/10 mcg, glimepiride, and sitagliptin showed significant reduction in HbA1c in both models. P-score ranking revealed saxagliptin+metformin, liraglutide+metformin, and liraglutide stayed the top three treatments. Moreover, there was no difference in the incidence of various adverse events versus placebo, except that the incidence of abdominal pain in liraglutide+metformin were significantly higher than placebo in additive model. This might be because the additive model assumed that the effect of the treatment combination is the sum of the effects of its components (22). Meanwhile, there were four unpublished clinical studies (NCT01204775, NCT01554618, NCT01434186, and NCT00658021) that did not provide specific baseline information (age, baseline HbA1c level, baseline weight, and baseline BMI) with participants, two were phase III studies on exenatide and two were phase III studies on saxagliptin+metformin. Therefore, although the efficacy and safety of saxagliptin+metformin were superior to other treatments in our analysis, further studies were needed to determine whether the treatment needed to meet a specific baseline level. We also performed sensitivity analyses of published studies, which showed that the ranking of the included treatments was consistent with the primary outcomes (**Supplemental Material S4**). Compared with metformin and exenatide–5/10 mcg, dapagliflozin, sitagliptin+metformin, and saxagliptin+metformin showed significant reduction in HbA1c in both models. Compared with liraglutide and exenatide–2 mcg, saxagliptin+metformin also showed significant reduction in HbA1c in both models. There was no significant difference in the safety of the different treatments in any of the comparisons (Figures a15–a18).

Metformin has been shown to be safe and effective for treatment of type 2 diabetes in pediatric patients (25). The most common adverse events associated with metformin are gastrointestinal disorders, including abdominal pain and diarrhea (32). For other drugs, Shyangdan D et al. (12) proposed that liraglutide is a useful addition to options for treating type 2 diabetes, being effective in reducing blood glucose, while avoiding hypoglycemia and weight gain. However, it has been pointed out that the addition of liraglutide to metformin for children and adolescents increased frequency of gastrointestinal disorders (15). Gottschalk M et al. (16) found that glimepiride reduced HbA1c similarly to metformin with greater weight gain. In comparison with previous studies, we compared treatments without head-to-head comparisons by integrating results from RCTs of different treatments. Through indirect comparison, we found that the result of liraglutide and its combination with metformin are in accordance with Shyangdan D et al. However, metformin showed significant reduction in HbA1c compared with glimepiride, which was inconsistent with direct comparisons. In addition, dapagliflozin, sitagliptin+metformin, and saxagliptin+metformin could significantly reduce HbA1c level for patients aged 10–17 years besides for the drugs approved by FDA and the incidence of abdominal pain or diarrhea was not significantly higher than metformin and liraglutide. These treatments deserved more extensive clinical trials to ensure their efficacy and safety, thus, providing better treatment options for children and adolescents with type 2 diabetes.

Furthermore, we also searched PubMed until September 2021 to identify pertinent analyses in adults with type 2 diabetes. Consistent with our findings in children, those studies have found that liraglutide combined with metformin is significantly better than metformin alone in reducing HbA1c, which is similar to glimepiride (33, 34). List JF et al. (35) showed that dapagliflozin had a greater ability to reduce HbA1c levels than metformin, with no significant difference in the incidence of adverse events. Aschner P et al. (36) demonstrated that sitagliptin was no significant in improving HbA1c compared with metformin, but a lower incidence of gastrointestinal disorders was observed in sitagliptin group. DeFronzo RA et al. (37) reported that saxagliptin+metformin was generally well tolerated and led to statistically significant reduction in HbA1c versus metformin+placebo. These results are in accordance with Pfützner A et al. (38), which compared saxagliptin+metformin with metformin alone.

More and more studies have focused on the effects of glucose-lowering drugs on cardiovascular function in type 2 diabetes adults. Tsapas A et al. (39) indicated that cardiovascular risk increased in patients receiving metformin-based background therapy, conversely, GLP-1 RAs, and SGLT-2 inhibitor have a favorable effect on certain cardiovascular outcomes. Since gastrointestinal disorders are of greater concern in adolescents than in adults, our study considered them as major factors in comparing treatments. Combining all efficacy outcomes, we found that DPP-4 inhibitor in combination with metformin (saxagliptin+metformin, sitagliptin+metformin) and GLP-1 RAs (exenatide and liraglutide) have a favorable effect. According to gastrointestinal disorders, although saxagliptin+metformin ranked lowest, there were no significant differences between treatments. So DPP-4 inhibitor in combination with metformin and GLP-1 RAs considered to be the better treatments in our study.

Our analysis did not combine different doses of drugs as a single treatment, so that we also obtained two comparisons of the same drugs at different doses, including exenatide and linagliptin. According to our results, exenatide–2 mcg ranks higher than exenatide–5/10 mcg in efficacy results. Blevins T et al. (40) conducted a study to compare effects of exenatide once weekly (ExQW) and exenatide twice daily (ExBID) on glycemic control, results indicated that ExQW produced significantly greater changes from baseline versus ExBID in HbA1c, similar to our findings. For linagliptin, since the data of different doses are all from the same study, the results obtained are consistent with the outcome of the original article (26).

To evaluate the evidence in a specific treatment situation, we performed subgroup analyses. The results of monotherapy group were almost similar to those of the primary outcomes. However, in combination group, there was no difference between any treatment and placebo. This might be because the sample size is limited.

Certain limitations should be acknowledged. First, there were two studies’ intervention duration less than 3 months (12 weeks), one is 5 weeks and the other was 8 weeks. Since HbA1c reflects the past 3 months’ blood glucose level, the duration of trials should be longer than 3 months. In order to ensure the sufficient statistical power, we did not exclude these two studies from the primary analysis. In addition, since multiple studies have included combinations, we have analyzed them using an additive network meta-analysis model, which may introduce bias due to the different dosing and allocation of combinations. Accordingly, we divided the study into monotherapy group and combination group for subgroup analysis. The results were consistent with the primary outcomes. Moreover, our analysis only considered four indicators to evaluate the efficacy, and some secondary indicators were missing. Some studies about hypoglycemic drugs analyzed the change of BMI and weight loss from baseline as outcomes, so further studies are needed to corroborate our conclusions. Meanwhile, although the efficacy and safety of saxagliptin+metformin were superior to other treatments in our analysis, due to the lack of baseline information, the treatment population of it needed to be further explored. Also, we did not compare different populations and age groups because of limited data. Finally, due to the limited number of studies and sample size, the consistency analysis could not be assessed.

## 5 Conclusions

According to the outcomes, the top 10 treatments of type 2 diabetes in children and adolescents aged 10–17 years were saxagliptin+metformin, liraglutide+metformin, liraglutide, dapagliflozin, exenatide–2 mcg, sitagliptin+metformin, linagliptin–5 mg, linagliptin–1 mg, metformin, and exenatide–5/10 mcg. Among them, liraglutide+metformin, sitagliptin+metformin, dapagliflozin, linagliptin–5mg, metformin, glimepiride, and sitagliptin were effective and safe for patients with BMI of 30–35 kg/m^2^ and weight of 80–90 kg. Due to the lack of baseline information, the treatment population targeted by saxagliptin+metformin needs to be further explored.

## Data availability statement

The original contributions presented in the study are included in the article/**Supplementary Material**. Further inquiries can be directed to the corresponding authors.

## Author contributions

SW conceived the study. SW, LH, and YTW designed the study. XL, YG and FQ selected the articles. SW, YH, and YTW appraised articles and extracted data for the clinical review. SW analyzed the data under the supervision of FX, YL, and HL, and wrote the first draft of the manuscript. MH provided clinical expertise in the analysis of the results. SW, YJ, YYY, YFY, QL, YF, YPF, JW, and YW, interpreted the data and contributed to the writing of the final version of the manuscript. All authors contributed to the article and approved the submitted version.

## Funding

This work was supported by the National Natural Science Foundation of China (Grant 82003557), Shandong Provincial Natural Science Foundation of China (ZR2019ZD02) and Shandong Province Key R&D Program (Science and Technology Demonstration Project) Project (2021SFGC0504).

## Acknowledgments

We are grateful to the supports of the National Natural Science Foundation of China, the National Key Research and Development Program of China, the Shandong Provincial Natural Science Foundation of China, and Shandong Provincial Key Research and Development project. We also thank the ClinicalTrials.gov (https://clinicaltrials.gov) for making the data publicly available.

## Conflict of interest

The authors declare that the research was conducted in the absence of any commercial or financial relationships that could be construed as a potential conflict of interest.

## Publisher’s note

All claims expressed in this article are solely those of the authors and do not necessarily represent those of their affiliated organizations, or those of the publisher, the editors and the reviewers. Any product that may be evaluated in this article, or claim that may be made by its manufacturer, is not guaranteed or endorsed by the publisher.
