# Supplementary material for: Comparative efficacy and safety of glucose-lowering drugs in children and adolescents with type 2 diabetes: A systematic review and network meta-analysis
Source: Front Endocrinol (Lausanne). 2022 Aug 11;13:897776. doi: 10.3389/fendo.2022.897776 (PMC9402979; doi:10.3389/fendo.2022.897776)
Supplement: Supplementary file 1 [file DataSheet_1.pdf]

Supplementary Material

1 Data Sources

PubMed

((((diabetes mellitus, type 2[MeSH Terms]) OR (type 2 diabetes[Title/Abstract]) OR (type 2 diabetes mellitus[Title/Abstract]) OR (Type 2 diabetes[Title/Abstract]) OR (NIDDM[Title/Abstract]) OR (MODY[Title/Abstract])) AND (((children[Title/Abstract]) OR (adolescents[Title/Abstract]) OR (pediatric[Title/Abstract]) OR (adolescent[Title/Abstract]))) AND ((clinicaltrial(Filter) OR meta-analysis(Filter) OR ("Randomized Controlled Trial"[pt] OR "Controlled Clinical Trial"[pt] OR "Pragmatic Clinical Trial"[pt] OR "Equivalence Trial"[pt] OR "Clinical Trial, Phase III"[pt] OR "Randomized Controlled Trials as Topic"[mh] OR "Controlled Clinical Trials as Topic"[mh] OR "Random Allocation"[mh] OR "Double-Blind Method"[mh] OR "Single-Blind Method"[mh] OR Placebos[Mesh:NoExp] OR "Control Groups"[mh] OR (random*[tiab] OR sham[tiab] OR placebo*[tiab]) OR ((singl*[tiab] OR doubl*[tiab]) AND (blind*[tiab] OR dumm*[tiab] OR mask*[tiab])) OR ((tripl*[tiab] OR trebl*[tiab]) AND (blind*[tiab] OR dumm*[tiab] OR mask*[tiab])) OR (control*[tiab] AND (study[tiab] OR studies[tiab] OR trial*[tiab] OR group*[tiab])) OR (Nonrandom*[tiab] OR "non random*"[tiab] OR "non-random*"[tiab] OR "quasi-random*"[tiab] OR quasirandom*[tiab]) OR allocated[tiab] OR (("open label"[tiab] OR "open-label"[tiab]) AND (study[tiab] OR studies[tiab] OR trial*[tiab])) OR ((equivalence[tiab] OR superiority[tiab] OR "non-inferiority"[tiab] OR noninferiority[tiab]) AND (study[tiab] OR studies[tiab] OR trial*[tiab])) OR ("pragmatic study"[tiab] OR "pragmatic studies"[tiab]) OR ((pragmatic[tiab] OR practical[tiab]) AND trial*[tiab]) OR ((quasiexperimental[tiab] OR "quasi-experimental"[tiab]) AND (study[tiab] OR studies[tiab] OR trial*[tiab])) OR (phase[ti] AND (III[ti] OR 3[ti]) AND (study[ti] OR studies[ti] OR trial*[ti])) OR (phase[ot] AND (III[ot] OR 3[ot]) AND (study[ot] OR studies[ot] OR trial*[ot]))) OR "systematic"[filter] OR "meta-analysis"[pt] OR "meta-analysis as topic"[mh] OR "meta analy*"[tw] OR metanaly*[tw] OR metaanaly*[tw] OR "met analy*"[tw] OR "integrative research"[tiab] OR "integrative review*"[tiab] OR "integrative overview*"[tiab] OR "research integration*"[tiab] OR "research overview*"[tiab] OR "collaborative review*"[tiab] OR "collaborative overview*"[tiab] OR "systematic review"[pt] OR "systematic reviews as topic"[mh] OR "systematic review*"[tiab] OR "technology assessment*"[tiab] OR "technology overview*"[tiab] OR "technology appraisal*"[tiab] OR "Technology Assessment, Biomedical"[mh] OR HTA[tiab] OR HTAs[tiab] OR "comparative efficacy"[tiab] OR "comparative effectiveness"[tiab] OR "outcomes research"[tiab] OR "indirect comparison*"[tiab] OR "Bayesian comparison"[tiab] OR (("indirect treatment"[tiab] OR "mixed-treatment"[tiab]) AND comparison*[tiab]) OR Cinahl*[tiab] OR "systematic overview*"[tiab] OR "methodological overview*"[tiab] OR "methodologic overview*"[tiab] OR "methodological review*"[tiab] OR "methodologic review*"[tiab] OR "quantitative review*"[tiab] OR "quantitative overview*"[tiab] OR "quantitative synthes*"[tiab] OR "pooled analy*"[tiab] OR Cochrane[tiab] OR Medline[tiab] OR Pubmed[tiab] OR Medlars[tiab] OR handsearch*[tiab] OR "hand search*"[tiab] OR "meta-regression*"[tiab] OR metaregression*[tiab] OR "data synthes*"[tiab] OR "data extraction"[tiab] OR "data abstraction*"[tiab] OR "mantel haenszel"[tiab] OR peto[tiab] OR "der-simonian"[tiab] OR dersimonian[tiab] OR "fixed effect*"[tiab] OR "multiple treatment comparison"[tiab] OR "mixed treatment meta-analys*"[tiab] OR "umbrella review*"[tiab] OR (("multiple paramet*"[tiab]) AND ("evidence synthesis"[tiab])) OR (("multi-

paramet*"[tiab]) AND ("evidence synthesis"[tiab])) OR ((multiparameter*[tiab]) AND ("evidence synthesis"[tiab])) OR "Cochrane Database Syst Rev"[Journal] OR "health technology assessment winchester, england"[Journal] OR "Evid Rep Technol Assess (Full Rep)"[Journal] OR "Evid Rep Technol Assess (Summ)"[Journal] OR "Int J Technol Assess Health Care"[Journal] OR "GMS Health Technol Assess"[Journal] OR "Health Technol Assess (Rockv)"[Journal] OR "Health Technol Assess Rep"[Journal])

Medline

(((TI=(adolescent)) OR TI=(children) OR TI=(pediatric)) AND (TI=(Type 2 Diabetes) OR TI=(diabetes mellitus, type 2) OR TI=(NIDDM) OR TI=(type 2 diabetSupplementary) OR TI=(type 2 diabetes))) AND ((DT==("ARTICLE" OR "Randomized Controlled Trial" OR "Controlled Clinical Trial" OR "Pragmatic Clinical Trial" OR "Clinical Study" OR "Adaptive Clinical Trial" OR "Equivalence Trial" OR "OTHER") AND SILOID==("MEDLINE")) NOT (DT==("REVIEW")))

Ovid

1. ((children or adolescents or pediatric) and type 2 diabetes).kw.
2. ((children or adolescents or pediatric) and diabetes mellitus, type 2).kw.
3. ((children or adolescents or pediatric) and type 2 diabetSupplementary).kw.
4. ((children or adolescents or pediatric) and type 2 diabetes).kw.
5. ((children or adolescents or pediatric) and NIDDM).kw.

Cochrane Central Register of Controlled Trials :

#1 MeSH descriptor: (Diabetes Mellitus, Type 2) this term only and with qualifier(s): (drug therapy - DT)
#2 children or adolescent or pediatric:ti,ab,kw in Trials
#3 #1 and #2 in Trials

ClinicalTrials.gov

The applied filters Studies With Results | Interventional Studies | Type 2 Diabetes | 18 years, Child | Phase 3, 4

2 Summary of bias risk

Three reviewers independently and in duplicate assessed the risk of bias in each trial for each outcome using the revised Cochrane Collaboration Risk of Bias tool RoB2.0. Domains assessed were the following:

1. Risk of bias arising from randomization process
2. Risk of bias due to deviations from intended interventions
3. Missing outcome data
4. Risk of bias in measurement of the outcome
5. Risk of bias in selection of the reported result

Overall risk-of-bias judgments were deemed as ‘low risk of bias’, ‘some concerns’ and ‘high risk of bias’ according to the tool algorithms. Any discrepancies between reviewers were resolved by consensus with a third investigator (Fig.a5).

3 Secondary Outcomes

3.1 Efficacy Outcomes

3.1.1 Reduction in FPG level

Nine studies involving 960 patients reported change in FPG from baseline. In fixed-effects network meta-analysis, compared to placebo, exenatide-2mcg (MD -21.70 [-23.10, -20.30]), sitagliptin+metformin (MD -14.40 [-15.12, -13.68]), metformin (MD -3.60 [-3.77, -3.43]), linagliptin-5mg (MD -1.89 [-2.63, -1.15]), liraglutide (MD -1.43 [-2.16, -0.70]), dapagliflozin (MD -0.94 [-1.36, -0.52]), exenatide-5/10mcg (MD -0.28 [-0.53, -0.03]) and all showed significant reduction in FPG.

In additive network meta-analysis, liraglutide, dapagliflozin, exenatide-2mcg, linagliptin-5mg, metformin and exenatide-5/10mcg showed the consistent results as the fixed-effects model, especially, sitagliptin+metformin (MD -8.17 [-8.69, -7.65]) showed less significant reduction (Fig.a6(A)). However, sitagliptin showed different result from the fixed-effects model (MD -4.57 [-5.06, -4.08] in fixed-effects model; MD 1.50 [0.81, 2.19] in additive model). This might be because the additive model assumed that the effect of the treatment combination is the sum of the effects of its components. Furthermore, exenatide-2mcg showed the greatest potential as the best intervention to improve FPG (P-score = 1.00 in both models) and sitagliptin+metformin was the second best (P-score = 0.89 in both models; Table S4).

3.1.2 Patients Achieving HbA1c Goals of < 7%

Eight studies involving 1,161 patients reported the percentage of patients achieving HbA1c goals of less than 7%. In fixed-effects network meta-analysis model, compared to placebo, liraglutide+metformin (OR 48.76 [12.02, 197.74]), sitagliptin+metformin (OR 27.23 [7.19, 103.02]), metformin (OR 16.20 [4.83, 54.36]), glimepiride (OR 12.88 [3.50,47.50]) and dapagliflozin (OR 10.34 [1.24, 86.02]) showed significant improvement in the percentage of HbA1c < 7%. The additive network meta-analysis showed the consistent results (Fig.a6(B)). Furthermore, according to P-score, all treatments in this analysis were better than placebo (Table S5)

3.1.3 Patients Achieving HbA1c Goals of ≤ 6.5%

Three studies involving 392 patients reported the percentage of patients achieving HbA1c goals of less than 6.5%. In both models, no treatments showed significant improvement in the percentage of HbA1c ≤ 6.5% (Fig.a6(C)). Furthermore, according to P-score, all treatments in this analysis were better than placebo (Table S6).

3.2 Safety Outcomes

3.2.1 Hyperglycemia

Seven studies involving 998 patients reported the number of patients with hyperglycemia. We also used OR to present the effect of treatments. Since 3 studies could not be included in the fixed-effects model for analysis, the fixed-effects model included 4 studies with a total of 381 participants, and the additive model included all 7 studies with a total of 998 participants. For all treatments in both models, there was no difference versus placebo in the incidence of adverse events (Fig.a7(A)). In

addition, dapagliflozin, sitagliptin+metformin, linagliptin-5mg, exenatide-2mcg, metformin, metformin+placebo, liraglutide+metformin were better than placebo (Table S7).

3.2.2 Hypoglycemia

Five studies involving 794 patients reported the number of patients with hypoglycemia, however, 2 studies could not enter the network. Finally we pooled 3 studies involving 312 patients to analyse. For all treatments in both models, there was no difference versus placebo in the incidence of hypoglycemia (Fig.a7(B), Table S8).

3.3.3 Gastrointestinal Disorders

Eight studies involving 1,085 patients reported the number of patients with upper abdominal pain. Since half of the studies were available for fixed-effects model, only additive models were used. For all treatments, there was no difference versus placebo in the incidence of upper abdominal pain (Fig.a7(C)). Furthermore, exenatide-2mcg showed better results than placebo (Table S9).

Ten studies involving 1,147 patients reported the number of patients with diarrhoea. Since 3 studies could not be included in the fixed-effects model for analysis, the fixed-effects model included 7 studies with a total of 530 participants, and the additive model included all studies with a total of 1,147 participants. For all treatments, there was no difference versus placebo in the incidence of diarrhoea (Fig.a7(D)). Furthermore, exenatide-2mcg, glimepiride, dapagliflozin and sitagliptin showed better results than placebo (Table S10).

Nine studies involving 907 patients reported the number of patients with vomiting. Since 1 study could not be included in the fixed-effects model for analysis, the fixed-effects model included 8 studies with a total of 773 participants, and the additive model included all studies with a total of 907 participants. For all treatments, there was no difference versus placebo in the incidence of vomiting (Fig.a7(E)). Furthermore, metformin, sitagliptin+metformin, liraglutide and sitagliptin showed better results than placebo (Table S11).

Seven studies involving 679 patients reported the number of patients with nausea. Since 1 study could not be included in the fixed-effects model for analysis, the fixed-effects model included 6 studies with a total of 545 participants, and the additive model included all studies with a total of 679 participants. For all treatments, there was no difference versus placebo in the incidence of nausea (Fig.a7(F)). Furthermore, metformin showed better results than placebo (Table S12).

Seven studies involving 979 patients reported the number of patients with abdominal pain. Since one study could not be included in the fixed-effects model for analysis, the fixed-effects model included 6 studies with a total of 845 participants, and the additive model included all 7 studies with a total of 979 participants. In additive network meta-analysis model, liraglutide+metformin (OR 7.84 [1.59, 38.67]) showed significant difference from placebo (Fig.a7(E)). Furthermore, exenatide-2mcg and saxagliptin+metformin showed better results than placebo (Table S13).

4 Sensitivity and Subgroup Analysis

4.1 Sensitivity analysis

4.1.1 Sensitivity analyses for only studies at low risk of bias.

HbA1c control

Nine studies involving 786 patients reported change in HbA1c from baseline. In fixed-effects network meta-analysis, compared to placebo, saxagliptin+metformin (MD -1.91% [-2.85%, -0.97%]), liraglutide (MD -0.90% [-1.35%, -0.45%]), sitagliptin+metformin (MD -0.89% [-1.04%, -0.73%]), exenatide-2mcg (MD -0.85% [-1.07%, -0.63%]), linagliptin-5mg (MD -0.64% [-1.08%, -0.20%]), metformin (MD -0.40% [-0.50%, -0.29%]), exenatide-5/10mcg (MD -0.27% [-0.45%, -0.09%]) and sitagliptin (MD -0.19% [-0.31%, -0.07%]) showed significant reduction in HbA1c (Fig.a8(A)). In additive network meta-analysis, all results were consistent with the fixed-effects model (Fig.a8(C)). Furthermore, saxagliptin+metformin also showed the greatest potential as the best intervention to improve HbA1c (P-score = 0.99 in both models) and liraglutide was the second best (P-score = 0.75 in fixed-effects model; 0.78 in additive model; Fig. a8(E)).

Adverse events

Nine studies involving 786 patients reported the percentage of patients with adverse events. We used OR to present the effect of treatments. For all treatments in both models, there was no difference versus placebo in the incidence of adverse events (Fig.a8(B), Fig.a8(D)). Furthermore, linagliptin-5mg (P-score = 0.76 in both models), dapagliflozin (P-score = 0.71 in fixed-effects model; 0.70 in additive model) and exenatide-2mcg (P-score = 0.65 in both models) showed better effects than placebo (Fig.a8(F)).

4.1.2 Sensitivity analysis with Bayesian network meta-analysis.

We fitted a Bayesian fixed effects network meta-analysis model to verify our results. In this model, we estimated MD of the effects and the associated 95% CIs using Markov chain Monte Carlo algorithms. All analyses were conducted using the gemtc package (version 1.0-1) in R, version 4.1.2 (The R Foundation). We used the package's default setting including noninformative prior distributions with 4 parallel chains, where each chain consists of 50 000 samples after a 100 000-sample burn-in. To evaluate and rank regimens, we calculated rank probabilities and the Surface Under the Cumulative Ranking (SUCRA). The SUCRA is a numerical summary that accounts for both magnitude and uncertainty of the estimated effect for each regimen. A larger SUCRA value indicates better performance for the outcome. We ranked treatments based on SUCRA with respect to each safety and efficacy outcome.

In Bayesian fixed effects network meta-analysis, compared to placebo, the reduction of HbA1c was significantly larger in saxagliptin+metformin (MD -2.20% [-2.80%, -1.60%]), liraglutide+metformin (MD -1.50% [-1.50%, -1.40%]), liraglutide (MD -0.90% [-1.00%, -0.75%]), sitagliptin+metformin (MD -0.89% [-0.95%, -0.83%]), dapagliflozin (MD -0.87% [-1.00%, -0.71%]), exenatide-2mcg (MD -0.85% [-0.97%, -0.73%]), linagliptin-5mg (MD -0.64% [-0.86%, -0.42%]), linagliptin-1mg (MD -0.48% [-0.76%, -0.20%]), metformin (MD -0.40% [-0.43%, -0.37%]), glimepiride (MD -0.33% [-0.36%, -0.30%]), exenatide-5/10mcg (MD -0.27% [-0.37%, -0.17%]) and sitagliptin (MD -0.19% [-0.24%, -0.14%]), respectively (Fig.a9(A)).

Furthermore, saxagliptin+metformin showed the greatest potential as the best intervention to improve HbA1c, liraglutide+metformin was the second best and liraglutide third(Fig.a9(B)).

4.1.3 Sensitivity analyses for only studies with BMI (30-35kg/m^2) and Weight(79.8-92.8kg).

HbA1c control

Seven studies involving 1005 patients reported change in HbA1c from baseline. In fixed-effects model, compared to placebo, the reduction of HbA1c was significantly larger in liraglutide_metformin (MD -1.46% [-1.65%, -1.27%]), sitagliptin_metformin (MD -0.89% [-1.05%, -0.73%]), dapagliflozin (MD -0.87% [-1.18%, -0.56%]), linagliptin-5mg (MD -0.64% [-1.08%, -0.20%]), metformin (MD -0.40% [-0.51%, -0.29%]), glimepiride (MD -0.25% [-0.37%, -0.13%]) and sitagliptin (MD -0.19% [-0.31%, -0.07%]) respectively (Fig.a10(A)). In additive network meta-analysis, all results were almost consistent with the fixed-effects model (Fig.a10(C)). The ranking of the treatments was similar to the primary outcomes (Fig.a10(E)).

Adverse events

Seven studies involving 1005 patients reported the percentage of patients with adverse events. For all treatments in both models, there was no difference versus placebo in the incidence of adverse events (Fig.a10(B), Fig.a10(D)). Furthermore, linagliptin-5mg (P-score = 0.77 in both models) and dapagliflozin (P-score = 0.71 in both models) showed better effect than placebo (Fig.a10(F)).

4.1.4 Sensitivity analyses for published studies.

HbA1c control

Eight studies involving 987 patients reported change in HbA1c from baseline. In fixed-effects model, compared to placebo, the reduction of HbA1c was significantly larger in liraglutide_metformin (MD -1.46% [-1.65%, -1.27%]), liraglutide (MD -0.90% [-1.35%, -0.45%]), sitagliptin_metformin (MD -0.89% [-1.05%, -0.73%]), dapagliflozin (MD -0.87% [-1.18%, -0.56%]), linagliptin-5mg (MD -0.64% [-1.08%, -0.20%]), metformin (MD -0.40% [-0.51%, -0.29%]), glimepiride (MD -0.25% [-0.37%, -0.13%]) and sitagliptin (MD -0.19% [-0.31%, -0.07%]) respectively (Fig.a11(A)). In additive network meta-analysis, all results were almost consistent with the fixed-effects model (Fig.a11(C)). The ranking of the treatments was similar to the primary outcomes (Fig.a11(E)).

Adverse events

Eight studies involving 987 patients reported the percentage of patients with adverse events. For all treatments in both models, there was no difference versus placebo in the incidence of adverse events (Fig.a11(B), Fig.a11(D)). Furthermore, linagliptin-5mg and dapagliflozin showed better effects than placebo (Fig.a11(F)).

4.2 Subgroup analysis

Since multiple studies have included combinations, although we have analyzed them using an additive network meta-analysis model, the analysis may introduce bias due to the different dosing and allocation of combinations. We found that all of the drugs compared in the study were monotherapy or monotherapy plus metformin, so in the subgroup analysis, all treatments were divided into monotherapy and combination groups and compared the results with previous results to determine whether they were consistent.

4.2.1 Monotherapy group

HbA1c control

Eight studies involving 867 patients reported change in HbA1c from baseline. In fixed-effects model, compared to placebo, the reduction of HbA1c was significantly larger in liraglutide (MD -0.90% [-1.35%, -0.45%]), dapagliflozin (MD -0.87% [-1.18%, -0.56%]), exenatide-2mcg (MD -0.85% [-1.07%, -0.63%]), linagliptin-5mg (MD -0.64% [-1.08%, -0.20%]), metformin (MD -0.40% [-0.50%, -0.29%]), exenatide-5/10mcg (MD -0.27% [-0.45%, -0.09%]), glimepiride (MD -0.25% [-0.37%, -0.13%]) and sitagliptin (MD -0.19% [-0.31%, -0.07%]) respectively (Fig.a12(A)). In additive network meta-analysis, all results are consistent with the fixed-effects model (Fig.a12(C)). The rank of the treatments were similar to the primary outcomes (Fig.a12(E)).

Adverse events

Seven studies involving 677 patients reported the percentage of patients with adverse events. For all treatments in both models, there was no difference versus placebo in the incidence of adverse events (Fig.a12(B), Fig.a12D)). Furthermore, linagliptin-5mg (P-score = 0.79 in both models), dapagliflozin (P-score = 0.73 in both models) and exenatide-2mcg (P-score = 0.68 in both models) showed better results than placebo (Fig.a12(F)).

4.2.2 Combination group

HbA1c control

Four studies involving 368 patients reported change in HbA1c from baseline. Contrary to the primary outcomes, there was no difference between any treatment and placebo (Fig.a13(A), Fig.a13(C)). This might be because the sample size is too limited. Nevertheless, saxagliptin+metformin still ranked higher than metformin in reducing HbA1c levels (Fig.a13(E)).

Adverse events

Four studies involving 368 patients reported the percentage of patients with adverse events. For all treatments in both models, there was no difference versus placebo in the incidence of adverse events (Fig.a13(B), Fig.a13(D)).The rank of the treatments were similar to the primary outcomes (Fig.a13(F)).

Table S1 Evaluation of heterogeneity in networks

Outcome	Cochran’s Q statistic
HbA1c	<0.001
FPG	< 0.001
Patients Achieving HbA1c Goals of < 7%	< 0.001
Patients Achieving HbA1c Goals of ≤6.5%	< 0.001
AE	<0.001
Hyperglycemia	< 0.001
Hypoglycemia	< 0.001
Abdominal pain	< 0.001
Diarrhoea	< 0.001
upper abdominal pain	< 0.001
Vomiting	< 0.001
Nausea	< 0.001

Table S2 Treatment Rank according HbA1c

	Fixed effects model	Additive model	Mean
treatment	P-score		
saxagliptin+metformin	0.98	0.98	0.98
liraglutide+metformin	0.93	0.93	0.93
liraglutide	0.69	0.81	0.75
dapagliflozin	0.68	0.68	0.68
exenatide1	0.67	0.67	0.67
sitagliptin+metformin	0.70	0.58	0.64
linagliptin5	0.52	0.51	0.52
linagliptin1	0.39	0.38	0.38
metformin	0.37	0.35	0.36
exenatide2	0.23	0.19	0.21
sitagliptin	0.14	0.28	0.21
glimepiride	0.20	0.15	0.17
placebo	0.00	0.00	0.00

exenatide1 = exenatide-2mcg; exenatide2 = exenatide-5/10mcg;
linagliptin1 = linagliptin-1mg; linagliptin5 = linagliptin-5mg.

Table S3 Treatment Rank according Adverse Events

	**Fixed effects model**	**Additive model**	**Mean**
treatment	P-score		
linagliptin5	0.76	0.76	0.76
dapagliflozin	0.71	0.70	0.70
exenatide1	0.65	0.65	0.65
placebo	0.64	0.62	0.63
sitagliptin+metformin	0.70	0.55	0.62
sitagliptin	0.46	0.65	0.56
metformin	0.52	0.52	0.52
glimepiride	0.49	0.48	0.49
saxagliptin+metformin	0.46	0.44	0.45
liraglutide+metformin	0.39	0.28	0.34
exenatide2	0.34	0.31	0.32
liraglutide	0.20	0.37	0.29
linagliptin1	0.18	0.16	0.17

exenatide1 = exenatide-2mcg; exenatide2 = exenatide-5/10mcg;
linagliptin1 = linagliptin-1mg; linagliptin5 = linagliptin-5mg.

Table S4 Treatment Rank according FPG

	**Fixed effects model**	**Additive model**	**Mean**
treatment	P-score		
exenatide1	1.00	1.00	1.00
sitagliptin+metformin	0.89	0.89	0.89
metformin	0.78	0.67	0.72
linagliptin5	0.64	0.53	0.59
liraglutide	0.56	0.45	0.50
dapagliflozin	0.45	0.34	0.39
sitagliptin	0.00	0.78	0.39
exenatide2	0.27	0.16	0.22
linagliptin1	0.27	0.16	0.21
placebo	0.14	0.03	0.08

exenatide1 = exenatide-2mcg; exenatide2 = exenatide-5/10mcg; linagliptin1 = linagliptin-1mg; linagliptin5 = linagliptin-5mg.

Table S5 Treatment Rank according 7%

	**Fixed effects model**	**Additive model**	**Mean**
treatment	P-score		
liraglutide+metformin	0.97	0.97	0.97
sitagliptin+metformin	0.85	0.85	0.85
metformin	0.67	0.67	0.67
dapagliflozin	0.59	0.59	0.59
glimepiride	0.58	0.57	0.58
exenatide1	0.42	0.42	0.42
sitagliptin	0.23	0.24	0.23
exenatide2	0.14	0.13	0.14
placebo	0.05	0.04	0.05

exenatide1 = exenatide-2mcg; exenatide2 = exenatide-5/10mcg

Table S6 Treatment Rank according 6.5%

	**Both model**
treatment	P-score
exenatide1	0.89
sitagliptin	0.53
exenatide2	0.41
placebo	0.17

exenatide1 = exenatide-2mcg; exenatide2 = exenatide-5/10mcg

Table S7 Treatment Rank according Hyperglycemia

	Fixed effects model	Additive model	Mean
treatment	P-score		
dapagliflozin	0.89	0.93	0.91
linagliptin5	0.67	0.79	0.73
exenatide1	0.64	0.76	0.70
placebo	0.46	0.64	0.55
sitagliptin+metformin	/	0.50	0.50
liraglutide+metformin	/	0.32	0.32
metformin	/	0.31	0.31
linagliptin1	0.23	0.40	0.31
sitagliptin	0.12	0.29	0.20
glimepiride	/	0.07	0.07

exenatide2 = exenatide-5/10mcg; linagliptin1 = linagliptin-1mg; linagliptin5 = linagliptin-5mg.

Table S8 Treatment Rank according Hypoglycemia

	**Both model**
treatment	P-score
placebo	0.69
sitagliptin	0.59
linagliptin1	0.54
exenatide1	0.36
linagliptin5	0.32

exenatide2 = exenatide-5/10mcg; linagliptin1 = linagliptin-1mg; linagliptin5 = linagliptin-5mg.

Table S9 Treatment Rank according upper abdominal pain

Additive model	
treatment	P-score
exenatide2	0.78
placebo	0.75
sitagliptin+metformin	0.66
dapagliflozin	0.56
sitagliptin	0.56
exenatide1	0.53
liraglutide+metformin	0.51
metformin	0.27
saxagliptin+metformin	0.22
glimepiride	0.17

exenatide1 = exenatide-2mcg; exenatide2 = exenatide-5/10mcg

Table S10 Treatment Rank according Diarrhoea

	**Both model**
treatment	P-score
glimepiride	0.84
exenatide2	0.80
sitagliptin	0.68
dapagliflozin	0.66
placebo	0.61
linagliptin5	0.56
liraglutide	0.50
exenatide1	0.40
metformin	0.37
sitagliptin+metformin	0.33
saxagliptin+metformin	0.31
linagliptin1	0.26
liraglutide+metformin	0.17

exenatide1 = exenatide-2mcg; exenatide2 = exenatide-5/10mcg;
linagliptin1 = linagliptin-1mg; linagliptin5 = linagliptin-5mg.

Table S11 Treatment Rank according Vomiting

	**Fixed effects model**	**Additive model**	**Mean**
treatment	P-score		
sitagliptin	0.62	0.82	0.72
sitagliptin+metformin	0.71	0.68	0.70
metformin	0.63	0.72	0.68
liraglutide	0.78	0.47	0.63
placebo	0.56	0.63	0.60
exenatide2	0.35	0.40	0.37
liraglutide+metformin	/	0.34	0.34
exenatide1	0.31	0.34	0.33
saxagliptin+metformin	0.29	0.32	0.30
dapagliflozin	0.25	0.28	0.27

exenatide1 = exenatide-2mcg; exenatide2 = exenatide-5/10mcg

Table S12 Treatment Rank according Nausea

	**Fixed effects model**	**Additive model**	**Mean**
treatment	P-score		
metformin	0.71	0.75	0.73
placebo	0.64	0.67	0.65
exenatide2	0.54	0.57	0.56
sitagliptin	0.52	0.55	0.54
liraglutide	0.46	0.47	0.47
exenatide1	0.44	0.46	0.45
liraglutide+metformin	/	0.34	0.34
dapagliflozin	0.18	0.19	0.19

exenatide1 = exenatide-2mcg; exenatide2 = exenatide-5/10mcg

Table S13 Treatment Rank according Abdominal pain

	**Fixed effects model**	**Additive model**	**Mean**
treatment	P-score		
exenatide1	0.87	0.89	0.88
saxagliptin+metformin	0.78	0.81	0.79
placebo	0.58	0.64	0.61
sitagliptin	0.51	0.57	0.54
sitagliptin+metformin	0.31	0.40	0.36
glimepiride	0.26	0.34	0.30
metformin	0.19	0.30	0.24
liraglutide+metformin	/	0.05	0.05

exenatide2 = exenatide-5/10mcg

Table S14 Test for Funnel Plot Asymmetry

	**HbA1c**	**Adverse Events**
Regression Test	p = 0.2735	p = 0.4955
Rank Correlation Test	p = 0.5435	p = 0.8334

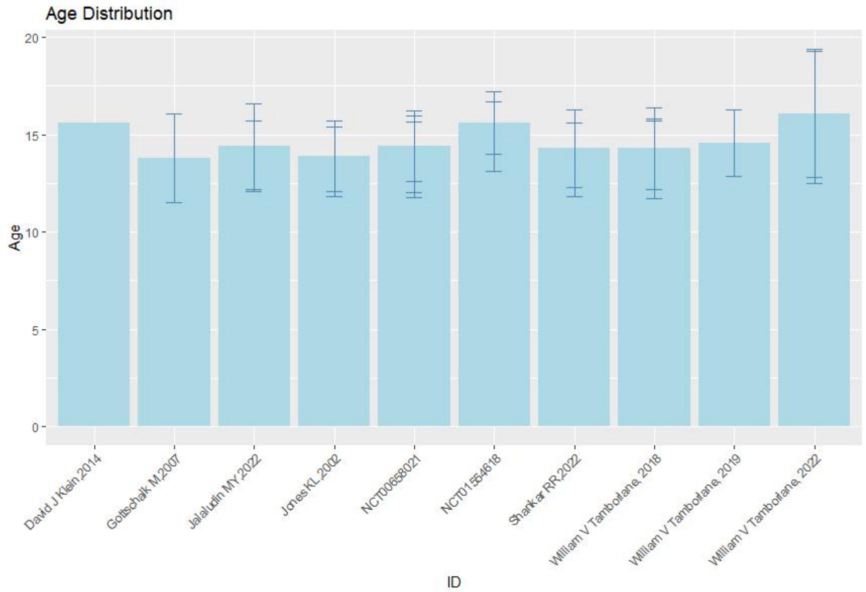

Fig.a1 Mean age of patients

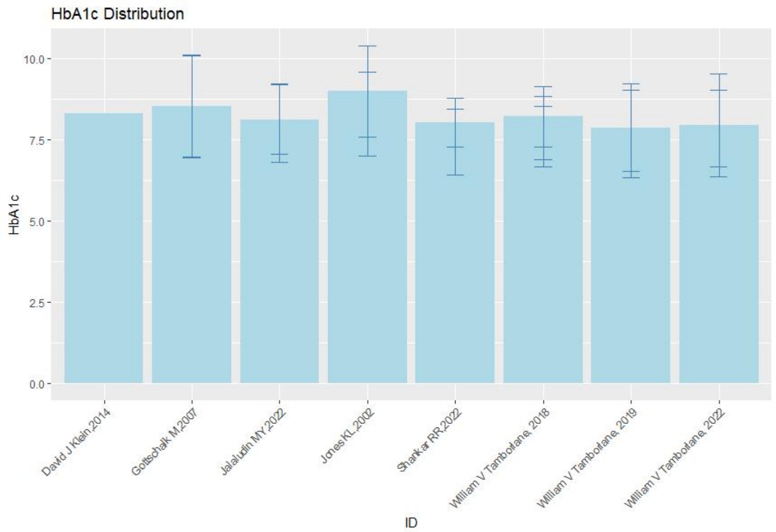

Fig.a2 HbA1c at baseline.

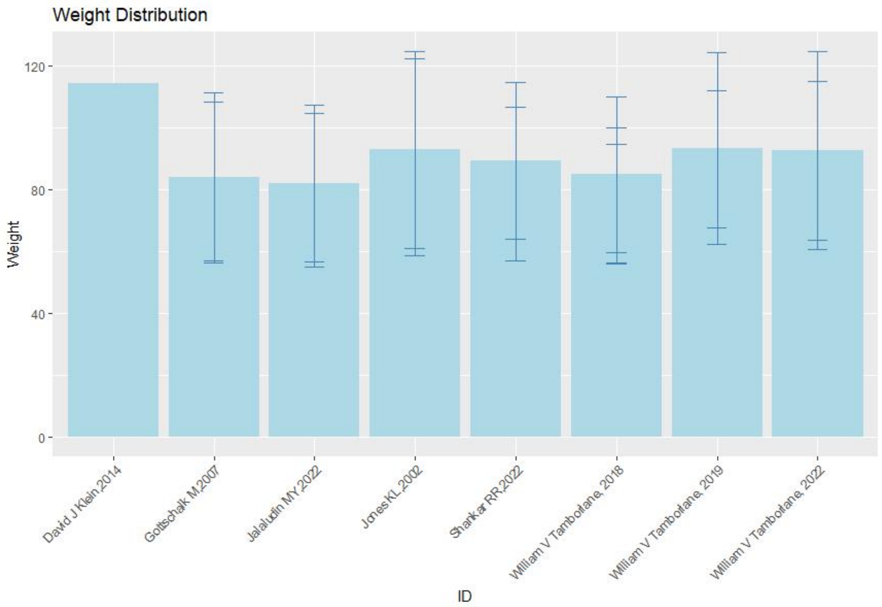

Fig.a3 Weight at baseline.

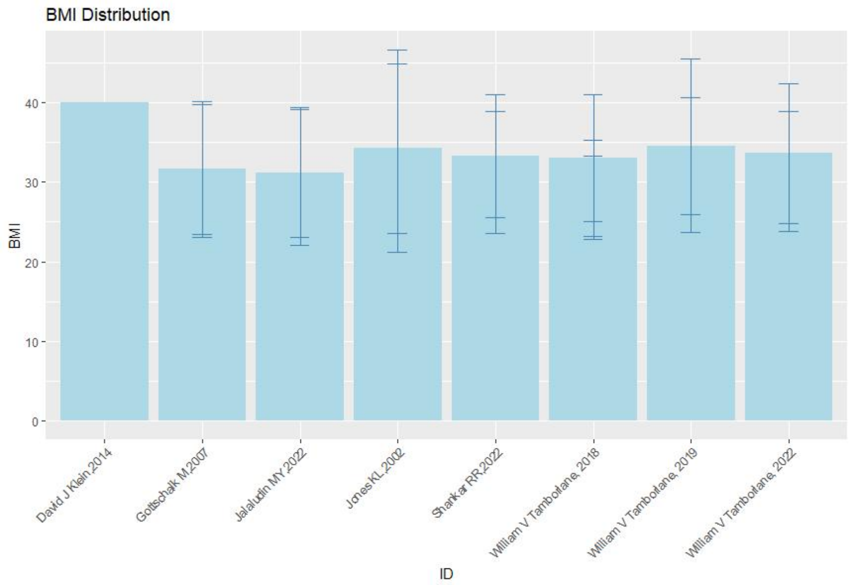

Fig.a4 BMI at baseline

Unique ID	Experimental	Comparator	Outcome	Weight	D1	D2	D3	D4	D5	Overall
NCT02725593	Dapagliflozin	placebo	HbA1c	1	+	+	!	+	+	!
NCT01554618	Exenatide	placebo	HbA1c	1	+	+	+	+	+	+
NCT01485614	Sitagliptin	placebo	HbA1c	1	+	+	+	+	+	+
NCT01434186	Saxagliptin+metformin	metformin	HbA1c	1	+	+	+	+	+	+
NCT01760447	Sitagliptin+metformin	metformin	HbA1c	1	+	+	+	+	+	+
NCT01204775	Saxagliptin+metformin	placebo	HbA1c	1	+	+	+	+	+	+
NCT00658021	Exenatide	placebo	HbA1c	1	+	+	+	+	+	+
David J Klein, 2014	liraglutide	placebo	HbA1c	1	+	+	+	+	+	+
Jones KL, 2002	metformin	placebo	HbA1c	1	+	+	+	+	+	+
Gottschalk M, 2007	glimepiride	metformin	HbA1c	1	+	!	+	+	+	!
William V Tamborlane, 2018	Linagliptin	placebo	HbA1c	1	+	+	+	+	+	+
Tamborlane WV, 2019	liraglutide+metformin	metformin	HbA1c	1	+	+	+	-	+	-

+ Low risk
! Some concerns
- High risk

D1 Randomisation process
D2 Deviations from the intended interventions
D3 Missing outcome data
D4 Measurement of the outcome
D5 Selection of the reported result

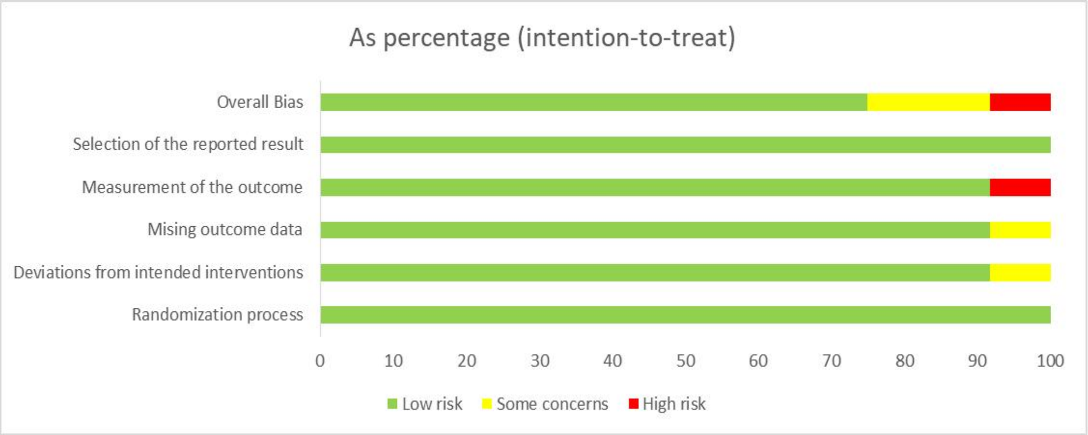

Fig.a5 Summary of bias risk

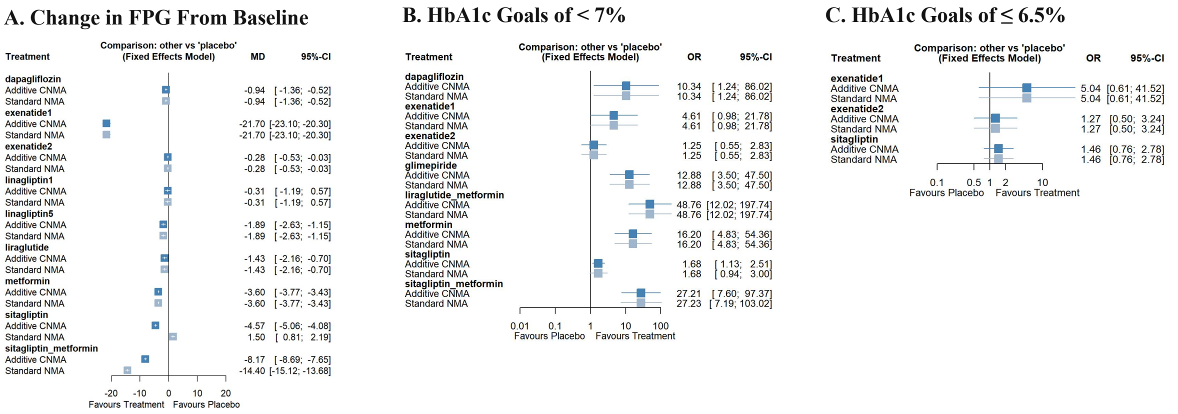

Fig. a6 Results for the Secondary outcomes of Efficacy compared with placebo.

Treatments are presented according to their effect estimate compared with placebo. Effect sizes are presented as MD or OR with 95% CIs. MD = mean difference; OR = odds ratio. exenatide1 = exenatide-2mcg; exenatide2 = exenatide-5/10mcg; linagliptin1 = linagliptin-1mg; linagliptin5 = linagliptin-5mg.

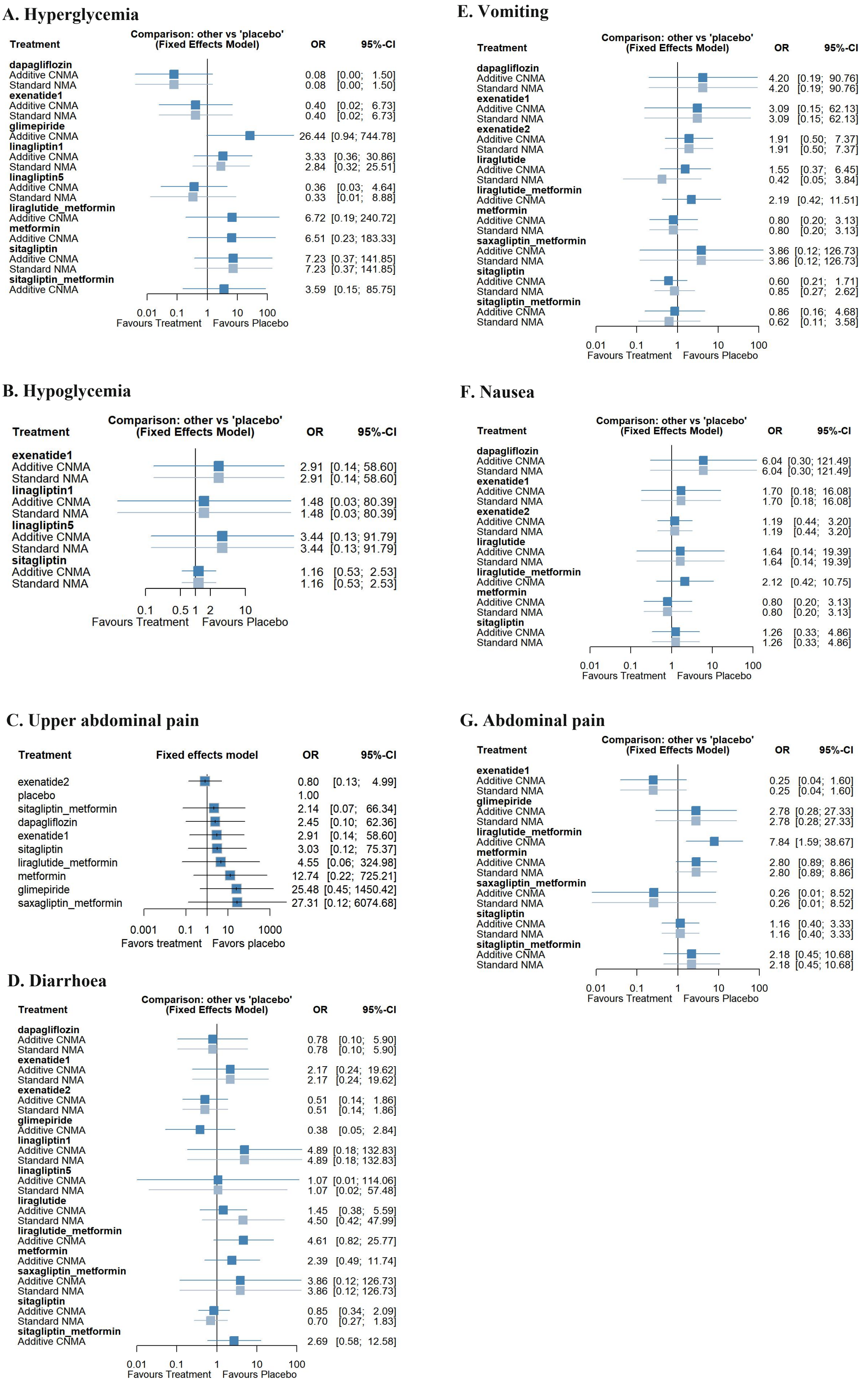

Fig. a7 Results for the Secondary outcomes of Safety compared with placebo.

Treatments are presented according to their effect estimate compared with placebo. Effect sizes are presented as OR with 95% CIs. OR = odds ratio. exenatide1 = exenatide-2mcg; exenatide2 = exenatide-5/10mcg; linagliptin1 = linagliptin-1mg; linagliptin5 = linagliptin-5mg.

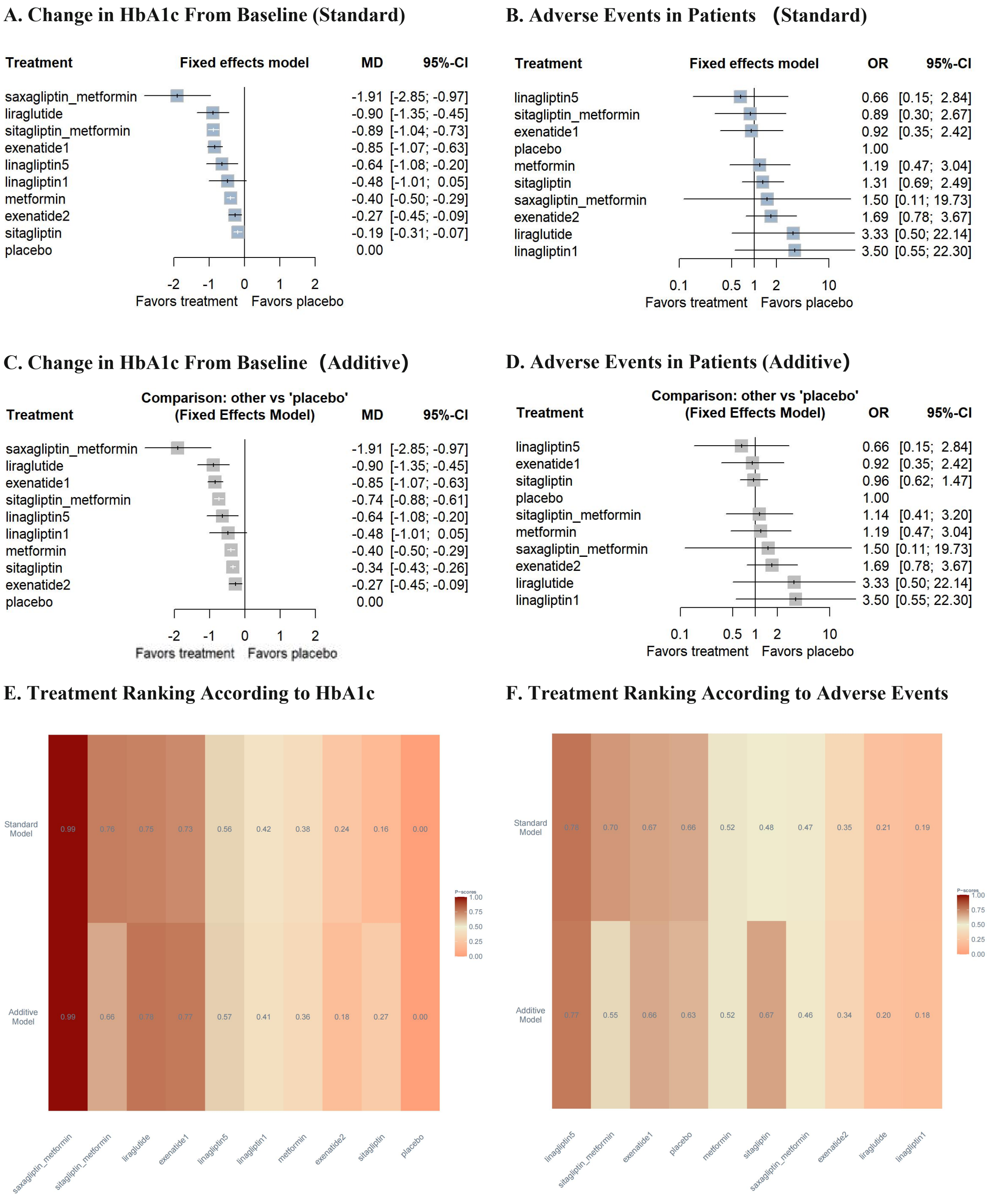

Fig. a8 Sensitivity analysis for studies with a low risk of bias.

Treatments are presented according to their effect estimate compared with placebo. Effect sizes are presented as MD or OR with 95% CIs. MD = mean difference; OR = odds ratio. exenatide1 = exenatide-2mcg; exenatide2 = exenatide-5/10mcg; linagliptin1 = linagliptin-1mg; linagliptin5 = linagliptin-5mg.

A. Change in HbA1c From Baseline (Standard)

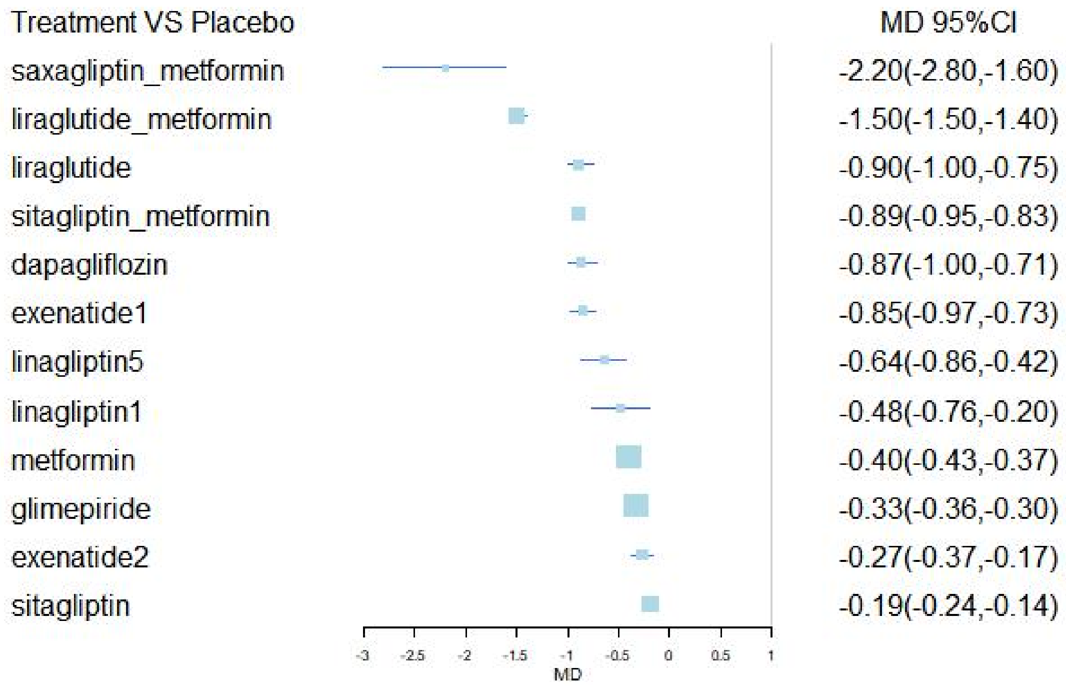

B. Treatment Ranking According to HbA1c

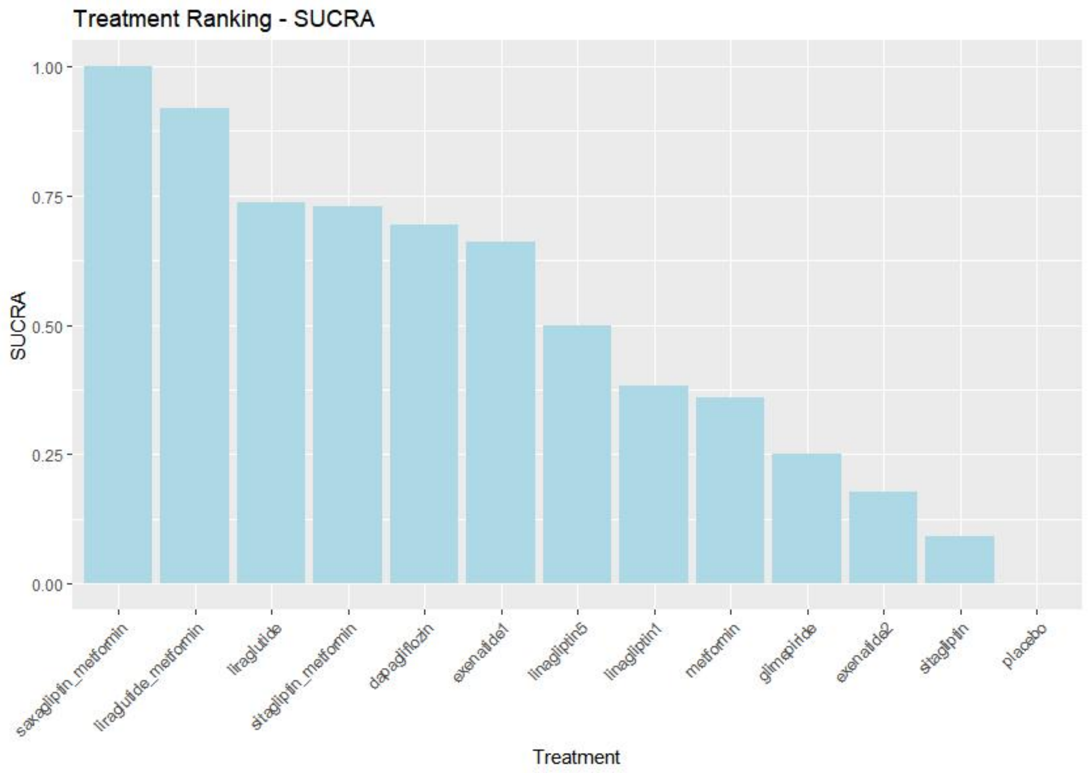

Fig. a9 Sensitivity analysis with Bayesian network meta-analysis.

Treatments are presented according to their effect estimate compared with placebo. Effect sizes are presented as MD with 95% CIs. MD = mean difference. exenatide1 = exenatide-2mcg; exenatide2 = exenatide-5/10mcg; linagliptin1 = linagliptin-1mg; linagliptin5 = linagliptin-5mg.

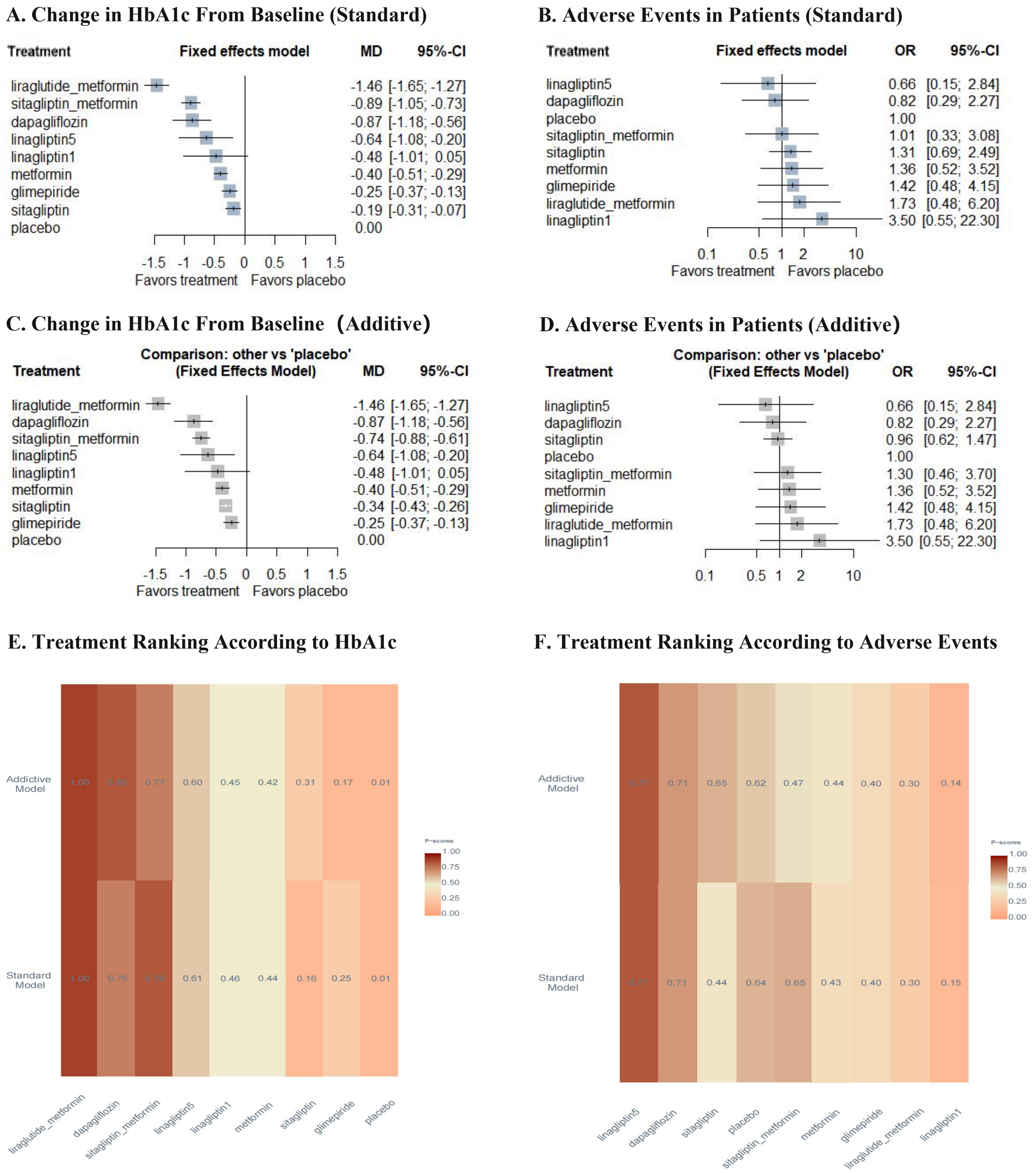

Fig. a10 Sensitivity analysis for BMI-Weight.

Treatments are presented according to their effect estimate compared with placebo. Effect sizes are presented as MD or OR with 95% CIs. MD = mean difference; OR = odds ratio. exenatide1 = exenatide-2mcg; exenatide2 = exenatide-5/10mcg; linagliptin1 = linagliptin-1mg; linagliptin5 = linagliptin-5mg.

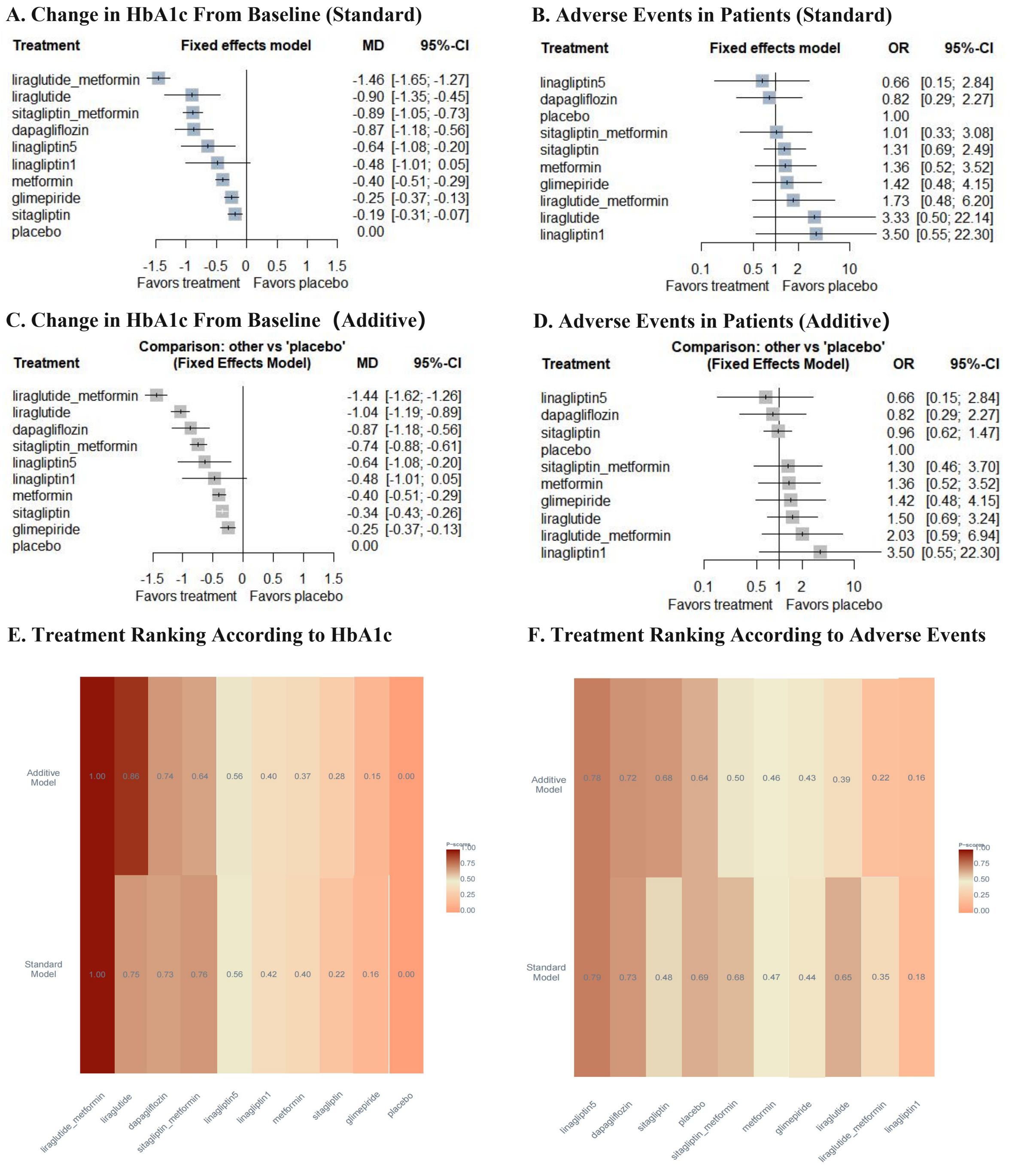

Fig. a11 Sensitivity analysis for published studies.

Treatments are presented according to their effect estimate compared with placebo. Effect sizes are presented as MD or OR with 95% CIs. MD = mean difference; OR = odds ratio. exenatide1 = exenatide-2mcg; exenatide2 = exenatide-5/10mcg; linagliptin1 = linagliptin-1mg; linagliptin5 = linagliptin-5mg.

A. Change in HbA1c From Baseline (Standard)

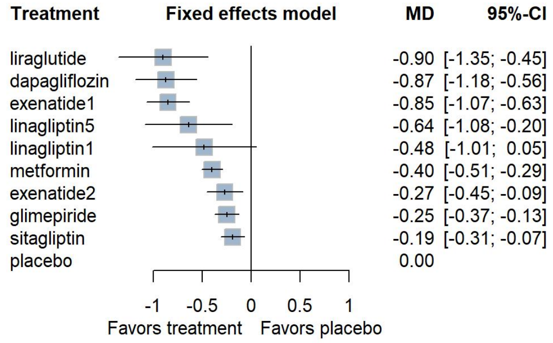

B. Adverse Events in Patients (Standard)

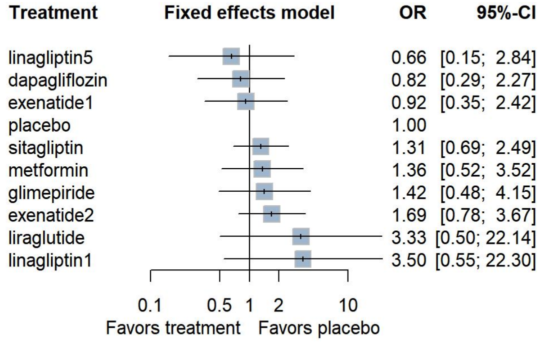

C. Change in HbA1c From Baseline (Additive)

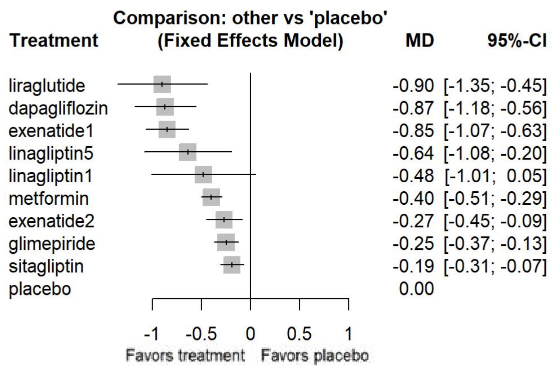

D. Adverse Events in Patients (Additive)

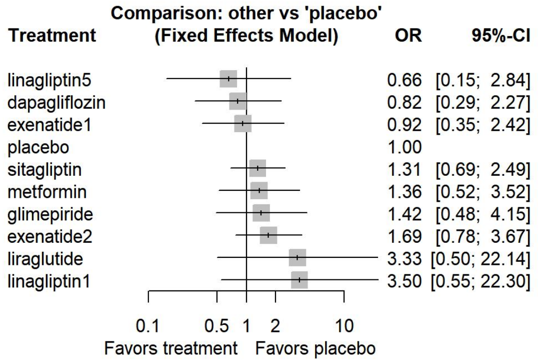

E. Treatment Ranking According to HbA1c

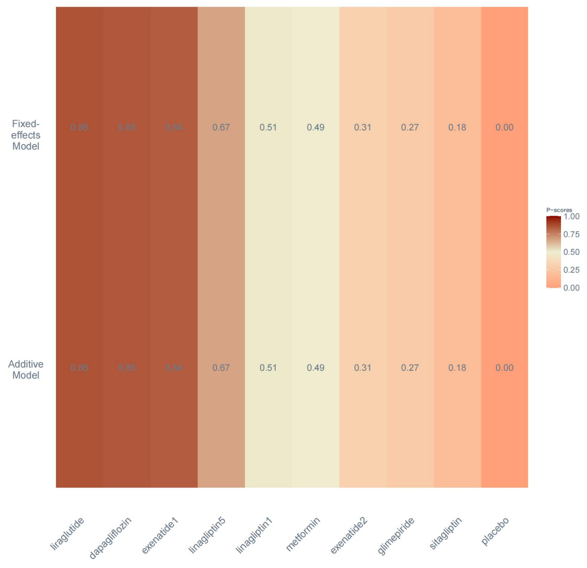

F. Treatment Ranking According to Adverse Events

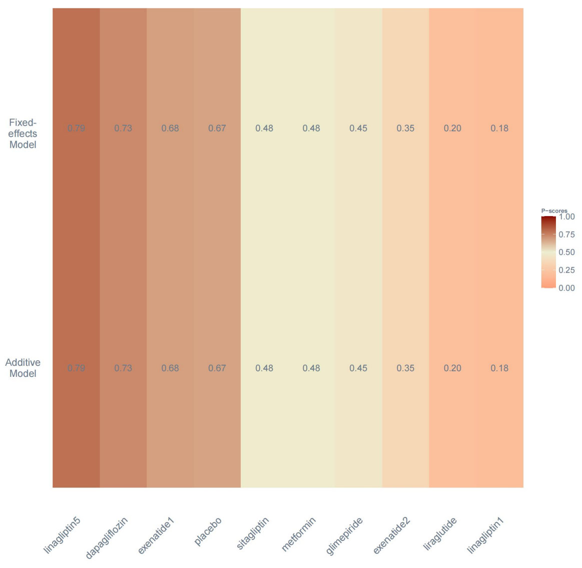

Fig. a12 Subgroup analysis for monotherapy group.

Treatments are presented according to their effect estimate compared with placebo. Effect sizes are presented as MD or OR with 95% CIs. MD = mean difference; OR = odds ratio. exenatide1 = exenatide-2mcg; exenatide2 = exenatide-5/10mcg; linagliptin1 = linagliptin-1mg; linagliptin5 = linagliptin-5mg.

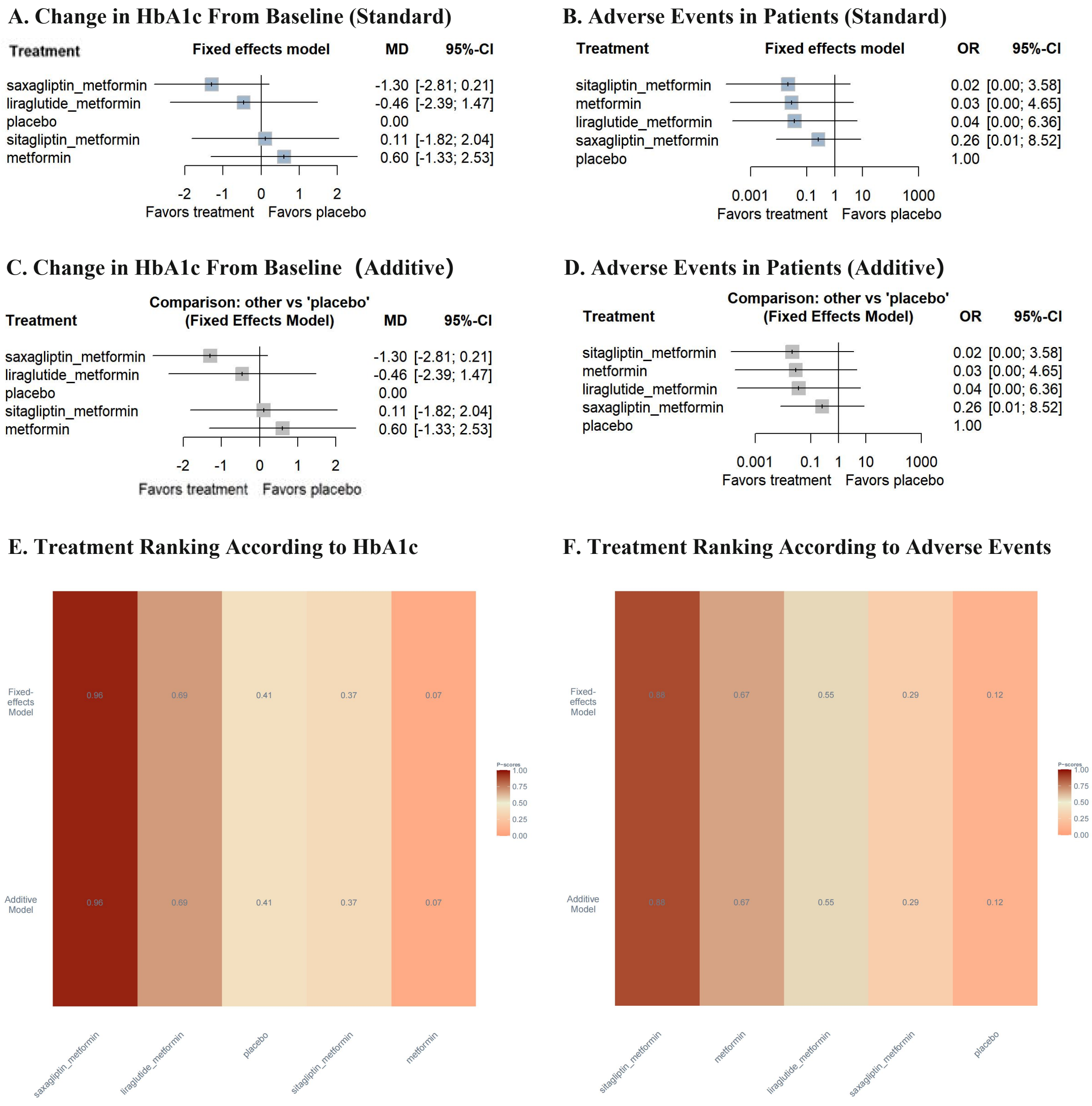

Fig. a13 Subgroup analysis for combination group.

Treatments are presented according to their effect estimate compared with placebo. Effect sizes are presented as MD or OR with 95% CIs. MD = mean difference; OR = odds ratio. exenatide1 = exenatide-2mcg; exenatide2 = exenatide-5/10mcg; linagliptin1 = linagliptin-1mg; linagliptin5 = linagliptin-5mg.

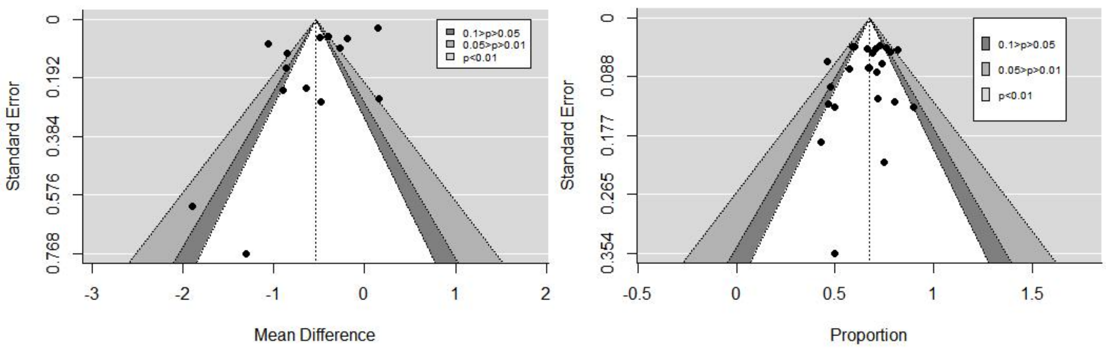

HbA1c **Adverse Events**

Fig.a14 Funnel plot

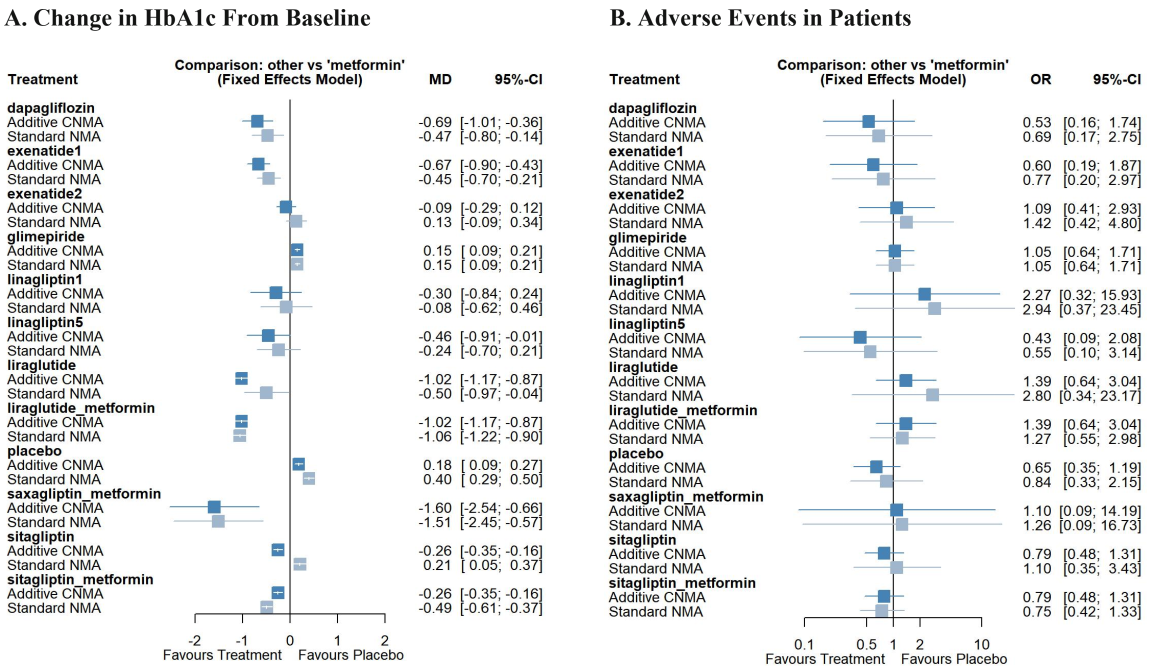

Fig.a15 Results for the primary outcomes compared with metformin.

Treatments are presented according to their effect estimate compared with placebo. Effect sizes are presented as MD or OR with 95% CIs. MD = mean difference; OR = odds ratio. exenatide1 = exenatide-2mcg; exenatide2 = exenatide-5/10mcg; linagliptin1 = linagliptin-1mg; linagliptin5 = linagliptin-5mg.

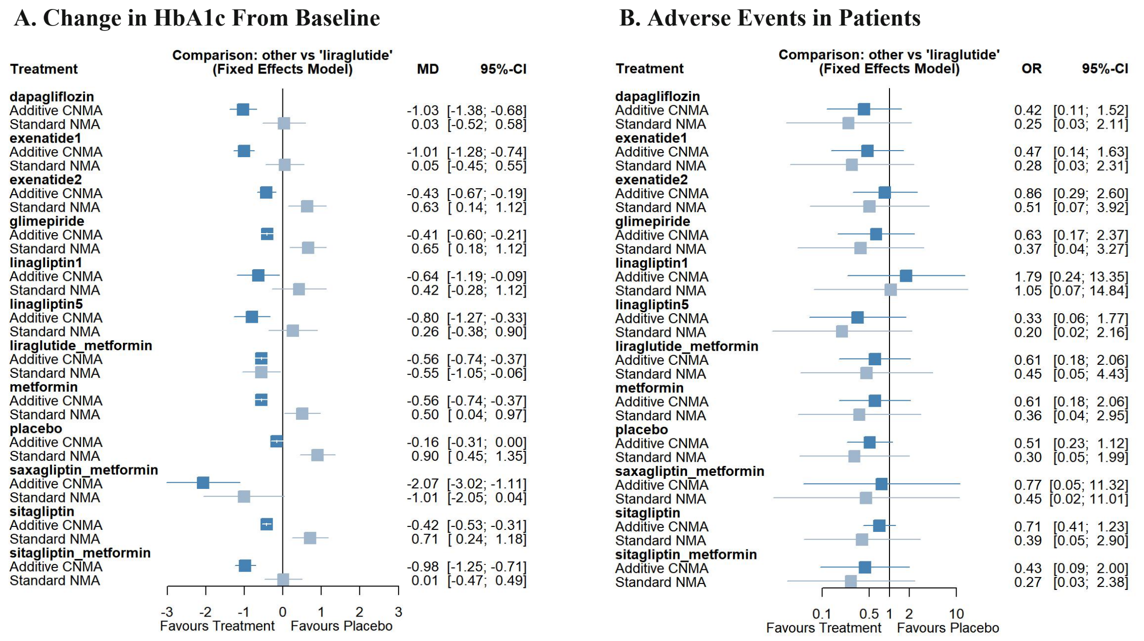

Fig. a16 Results for the primary outcomes compared with liraglutide.

Treatments are presented according to their effect estimate compared with placebo. Effect sizes are presented as MD or OR with 95% CIs. MD = mean difference; OR = odds ratio. exenatide1 = exenatide-2mcg; exenatide2 = exenatide-5/10mcg; linagliptin1 = linagliptin-1mg; linagliptin5 = linagliptin-5mg.

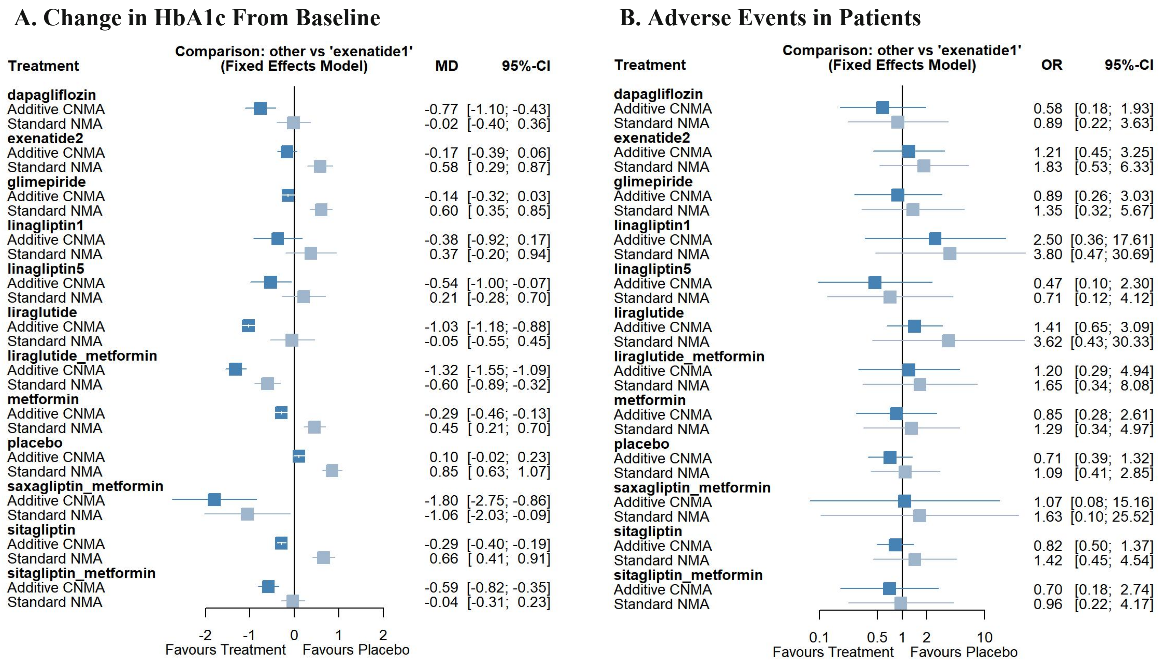

Fig. a17 Results for the primary outcomes compared with exenatide1.

Treatments are presented according to their effect estimate compared with placebo. Effect sizes are presented as MD or OR with 95% CIs. MD = mean difference; OR = odds ratio. exenatide1 = exenatide-2mcg; exenatide2 = exenatide-5/10mcg; linagliptin1 = linagliptin-1mg; linagliptin5 = linagliptin-5mg.

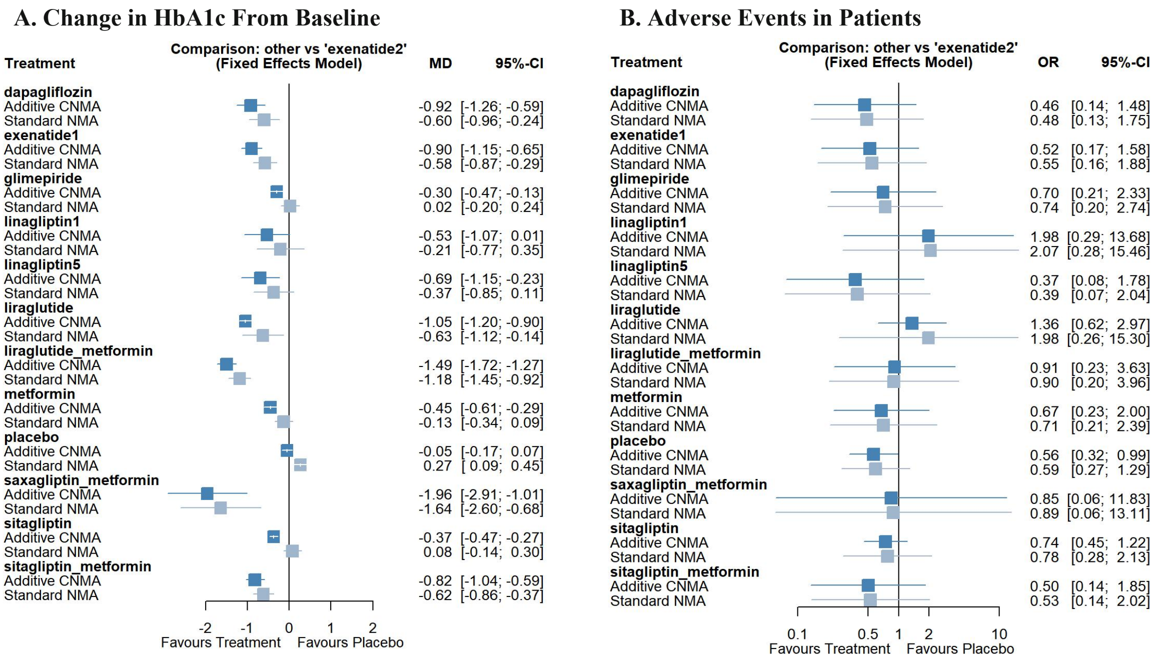

Fig. a18 Results for the primary outcomes compared with exenatide2.

Treatments are presented according to their effect estimate compared with placebo. Effect sizes are presented as MD or OR with 95% CIs. MD = mean difference; OR = odds ratio. exenatide1 = exenatide-2mcg; exenatide2 = exenatide-5/10mcg; linagliptin1 = linagliptin-1mg; linagliptin5 = linagliptin-5mg.
